# Reconstruction of protein domain evolution using single-cell amplified genomes of uncultured choanoflagellates sheds light on the origin of animals

**DOI:** 10.1098/rstb.2019.0088

**Published:** 2019-10-07

**Authors:** David López-Escardó, Xavier Grau-Bové, Amy Guillaumet-Adkins, Marta Gut, Michael E. Sieracki, Iñaki Ruiz-Trillo

**Affiliations:** 1 Institut de Biologia Evolutiva (CSIC-Universitat Pompeu Fabra), Passeig Marítim de la Barceloneta 37-49, 08003 Barcelona, Catalonia, Spain; 2 Institut de Ciències del Mar (ICM-CSIC), Passeig Marítim de la Barceloneta 37-49, 08003 Barcelona, Catalonia, Spain; 3 Departament de Genètica, Microbiologia i Estadística, Universitat de Barcelona, 08028 Barcelona, Catalonia, Spain; 4 Department of Vector Biology, Liverpool School of Tropical Medicine, Pembroke Place, Liverpool, L3 5QA, UK; 5 CNAG-CRG, Centre for Genomic Regulation (CRG), Barcelona Institute of Science and Technology (BIST), 08028 Barcelona, Spain; 6 Universitat Pompeu Fabra (UPF), 08003 Barcelona, Spain; 7 National Science Foundation, Arlington, VA 22314, USA; 8 ICREA, Pg. Lluís Companys 23, 08010 Barcelona, Spain

**Keywords:** protein domain evolution, choanoflagellates, animal multicellularity, single-cell genomics

## Abstract

Understanding the origins of animal multicellularity is a fundamental biological question. Recent genome data have unravelled the role that co-option of pre-existing genes played in the origin of animals. However, there were also some important genetic novelties at the onset of Metazoa. To have a clear understanding of the specific genetic innovations and how they appeared, we need the broadest taxon sampling possible, especially among early-branching animals and their unicellular relatives. Here, we take advantage of single-cell genomics to expand our understanding of the genomic diversity of choanoflagellates, the sister-group to animals. With these genomes, we have performed an updated and taxon-rich reconstruction of protein evolution from the Last Eukaryotic Common Ancestor (LECA) to animals. Our novel data re-defines the origin of some genes previously thought to be metazoan-specific, like the POU transcription factor, which we show appeared earlier in evolution. Moreover, our data indicate that the acquisition of new genes at the stem of Metazoa was mainly driven by duplications and protein domain rearrangement processes at the stem of Metazoa. Furthermore, our analysis allowed us to reveal protein domains that are essential to the maintenance of animal multicellularity. Our analyses also demonstrate the utility of single-cell genomics from uncultured taxa to address evolutionary questions.

This article is part of a discussion meeting issue ‘Single cell ecology’.

## 1. Introduction

Metazoa is the eukaryotic kingdom with most described species so far, around 1.3 million [1], and it is the multicellular group of eukaryotes for which the most differential cell types have been described [2]. Animals' success might be tightly linked to their multicellular complexity, which has been considered unique in the eukaryotic world [3], compared to other multicellular eukaryotic transitions [3,4]. Thus, the uniqueness of animal multicellularity raises the question of which mechanisms shaped the emergence of such special types of organisms from a unicellular ancestor more than 600 million years ago [4,5].

To address this question, efforts have been made, over the past decade, to reconstruct the Urmetazoan genomic content by comparing the genomic sequences of a broad and diverse spectrum of animals [6–8] with the genomes of their closest unicellular relatives: the Choanoflagellatea [9,10], Filasterea [11,12] and Teretosporea, which include ichthyosporeans and *Corallochytium limacisporum* [13,14]. These unicellular lineages and metazoans conform the Holozoa clade. Holozoa, together with Fungi and their unicellular relatives compose the eukaryotic supergroup known as Opisthokonta [15]. Thus, understanding the evolution of opisthokonts is critical to address the origin of animals.

The first genomic comparisons between animals and their unicellular relatives at a genomic level revealed a complex pre-metazoan genetic toolkit, already equipped with a rich repertoire of genes involved in multicellular functions. These included developmental transcription factors (like *Brachyury*, MYC, Runx, or P53), cell adhesion proteins (ECM elements, integrins, cadherins, and C-type lectins) and cell signalling receptors and transducers [9,10,16–20]. These findings suggested that co-option of ancestral genes into new functions was an important mechanism that occurred in the transition from the unicellular ancestor of animals to the Urmetazoa [20]. However, it was also found that not all the components of many animal signalling pathways, like the Hippo pathway, had a pre-metazoan origin. In some cases, only some ligands or receptors were present before the emergence of multicellularity. Those ancestral ligands and receptors were later on putatively co-opted in functioning within these animal signalling pathways [20,21]. Thus, the acquisition of new genes might have also played an important role in the emergence of animal multicellularity.

In two recent studies aiming at better reconstructing the Urmetazoan genome, bursts of new genic innovation were shown at the stem of Metazoa. One of those studies focused on the search of animal genetic innovations using a new method to infer homology [22], while the other expanded the genomic information of choanoflagellates by sequencing the transcriptomes of 19 choanoflagellate species [23]. Both studies claimed that approximately 1500–1700 genes were acquired during the transition towards animal multicellularity (three times more gene acquisition than has been reported in their unicellular ancestors). In particular, the most conserved animal-specific genes in extant metazoans were genes related to major signalling pathways such as components of TGF-beta or Wnt signalling pathways, and transcription factors like ETS or POU [22]. However, the results also showed genes that had probably been overlooked for their potential role in the emergence of animal multicellularity (like CEPB proteins), or even genes of unknown function [22,23]. Moreover, 372 genes previously thought to be metazoan-specific were shown to have originated in the Choanozoan (Choanoflagellates + animals) clade. These included genes related to animal innate immunity such as Toll-like receptors (TLRs) or its downstream signalling target NF-kappaB [23]. Thus, a broader taxon sampling is critical to have a complete view of the genetic and genomic changes that predated the transition towards animal multicellularity.

Choanoflagellates are a diverse protist group, with approximately 250 described species [24,25]. Molecular phylogenies based on a few genes have shown that choanoflagellates are divided into two major clades: Craspedida and Acanthoecida [24,26,27]. Craspedida includes the choanoflagellates with organic coverings that can be thecated (Salpingoecidae morphology) or non-thecated with non-restrictive coverings like glycocalyx or sheath (Codosigidae morphology) [28]. On the other hand, Acanthoecida is composed by choanoflagellates with a siliceous loricae, being most of the described species marine and with a tectiform lorica (around 150 species) [24], although there are 5-6 species described with nudiform lorica [25]. Furthermore, there are other clades of choanoflagellates, such as Clade L [29], FRESCHOs and MACHOs [30], that have been defined only by environmental sequences (18S rDNA gene). Thus, there is a vast hidden choanoflagellate diversity, which is uncultured and may be relevant to address animal origins.

In this work, we aim to improve this view by sequencing four single-cell amplified genomes (SAGs) of uncultured choanoflagellates belonging to distinct taxa and collected during the TARA Oceans expedition [31]. With the novel genomic information, we perform a new, taxon-rich phylogenomic analysis of the opisthokonts. Moreover, we reconstruct the evolutionary history of protein domains from the last eukaryotic common ancestor (LECA) to animals. Protein domains are the basic building blocks that determine the structure and the function of the proteins [32]. Thus, understanding the gains/losses of protein domains is crucial to better understand the genomic changes that mediated the transition towards animal multicellularity [33]. Also, reconstruction of protein domains is the most reliable method to get informative evolutionary insights when using single-cell genomics data, in which genomes appear fragmented and with low completeness values [34].

Our protein domain reconstruction analysis shows that, contrary to previous genetic reconstructions, the Metazoa ancestor did not suffer an important acquisition of new protein domains. This suggests that the genome innovation at the stem of Metazoa was mainly driven by duplications and protein domain shuffling processes. In addition, our analysis reveals key essential protein domains for animal functions and redefines the origin of some genes thought to be metazoan-specific, which appeared earlier in the unicellular ancestor of animals like the POU transcription factor.

## 2. Methods

### (a) Cell collection and whole genome amplification

**Figure 1.**
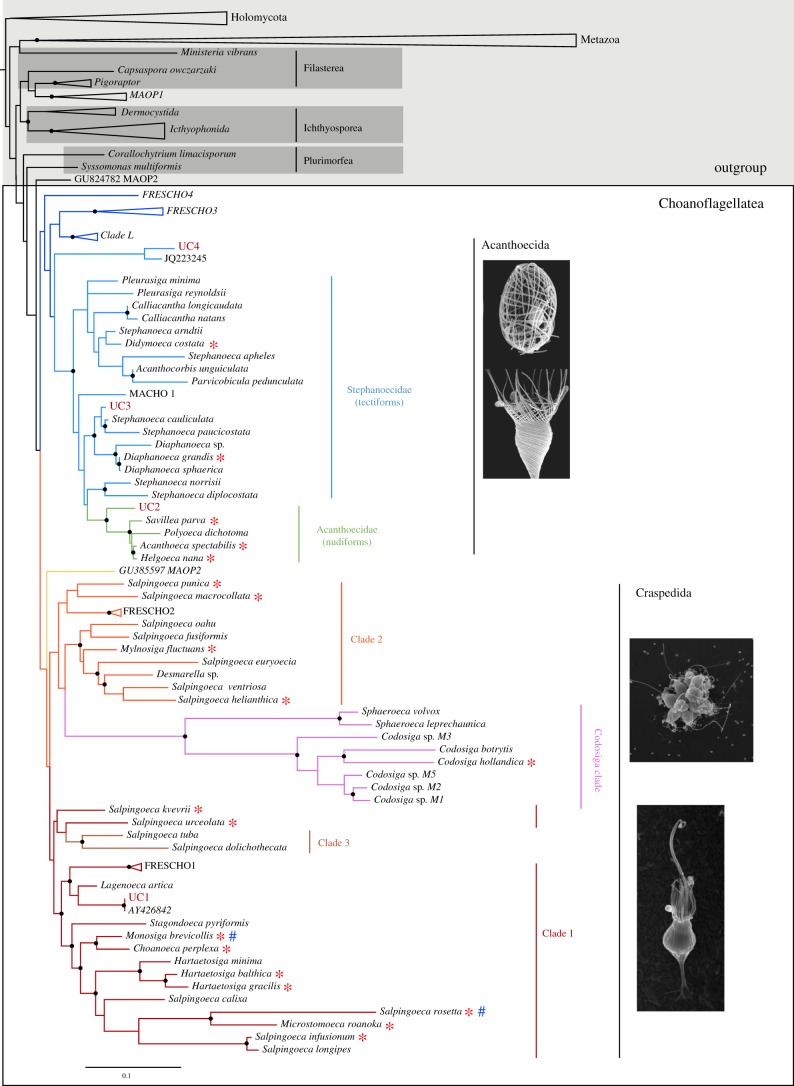
Phylogenetic position of the new choanoflagellate SAGs. Phylogenetic tree based on 117 sequences of the 18S rDNA gene, representing all that is known of the molecular diversity of choanoflagellates and unicellular holozoans, including environmental lineages. The phylogenetic analysis was inferred by maximum likelihood under the GTR+ Γ with IQ-TREE. Clades marked by a bullet (•) present high statistical split support, with values greater than 80% of SH-aLRT (bootstraps of single branch test) and greater than 95% of ultrafast bootstrap. Both indexes were computed with IQ-TREE. The remaining split supports obtained can be found at Figshare (https://doi.org/10.6084/m9.figshare.7819571.v1) in the tree file. The order and class names given are based on [30,41,42]. Choanoflagellates with transcriptomic data available are depicted with a red asterisk, and those with genomic data available are depicted with a blue hash. Choanoflagellates' craspedidan clades were named according to our phylogenomic analysis (figure 3). Clade 3 nomenclature and nomenclature within Acanthoecida are the same as in [27]. The Acanthoecida picture was taken from [28] and Craspedida pictures were taken in Nicole's King laboratory.

Cells for single-cell genomics were collected from the Mediterranean sea and different places in the Indian Ocean during the Tara Oceans expedition [35] and cryopreserved as described before [36]. Flow cytometry cell sorting, single cell lysis and whole genome amplification by Multiple Displacement Amplification (MDA) [37] were performed at the Bigelow Single-cell genomics facility (Boothbay, Maine, USA), as previously described [38–40]. The SAGs obtained were screened by PCR using universal eukaryotic 18S, as in previous studies [34,40]. Four SAGs were placed in distant phylogenetic positions compared to choanoflagellates for which there are available transcriptomic or genomic data (figure 1). Thus, they were deemed worthy of further analysis. Associated environmental data are summarized in electronic supplementary material, table S1, and further details can be found in PANGAEA [31,43].

### (b) Library preparation and genome sequencing

Four SAGs (UC1, UC2, UC3 and UC4) were sent for sequencing at CNAG (Barcelona, Spain). The libraries were constructed with the TruSeq Nano DNA Library Preparation Kit according to the manufacturer's protocol. Briefly, aiming for an insert size of 550 bp, 200 ng of gDNA were sheared by sonication using Covaris E210 (Covaris). Fragmented DNA was purified with Agencourt AMPure XP beads. Afterwards, end repair and size selection were performed, following 3′ adenylation reaction and ligation of the Illumina adapter indexes. DNA fragments were enriched by eight cycles of PCR and then purified with Agencourt AMPure XP beads. The Agilent Technologies 2100 Bioanalyzer DNA 1000 assay was used for library quality control and quantification.

Each library was sequenced using one lane of MiSeq reagent kit v2 (Illumina). The sequencing run was performed according to standard Illumina operation procedures in paired-end mode, with a read length of 2 × 251 bp and a yield of greater than 11 Gb. Primary data analysis, image analysis, base calling and quality scoring of the run were processed using the manufacturer's software Real Time Analysis (RTA 1.18.54) and followed by generation of FASTQ sequence files by CASAVA v. 1.8. The reads obtained were used to perform a downsampling analysis as described in [34]; UC1 and UC4 presented longer assemblies and the curve was still not saturated. Thus, we decided to apply more sequencing depth for them. Finally, UC1 and UC4 were sequenced in 3 Miseq lanes with a yield of greater than 34 Gb. Raw reads can be found at ENA (European Nucleotide Archive: ebi.ac.uk/ena), project accession number PRJEB31614.

### (c) Genome assembly and annotation

Raw reads obtained were trimmed with Trimmomatic v. 3.0 [44] using the following options: ILLUMINACLIP:/adapters/NexteraPE-PE.fa:2:40:15 HEADCROP:10 CROP:240 SLIDINGWINDOW:6:20 MINLEN:50. A range of between 42 and 45 million reads was obtained from the low-quality SAGs UC2 and UC3, and for the SAGs with more sequencing applied—UC1 and UC4—the range moves between 110 and 125 million reads (electronic supplementary material, table S2). Next, we performed the genome assembly with SPAdes v3.6.1 [45] with the options --sc, --careful and -k 21,33,55,77,99. The final genome statistics were obtained with QUAST [46]. The percentage of core eukaryotic conserved proteins was calculated with CEGMA [47] and BUSCO [48]. We screened for mitochondrial genomic sequences in our SAGs by performing a tBLASTn v. 2.2.31+ [49] using as query the mitochondrial proteins of *Andalucia godoyi* [50] with a cut-off e-value of 1e-04. Only the SAG UC2 presented three scaffolds with mitochondrial proteins. We mapped SAG UC2 reads over the scaffolds selected using the program Bowtie2 v. 21.0 [51] in order to perform a re-assembly with SPAdes v. 3.6.1 [45], and to see if we could recover in one scaffold the full mitochondrial genomic sequence. The assembly yielded a more fragmented output, and two of the previously selected scaffolds contained proteins that came from bacterial contamination. Thus, we decided to keep the first scaffolds obtained (32 Kb length). UC2 partial mitochondrial genome is available at Figshare (https://doi.org/10.6084/m9.figshare.7819571.v1). We annotated the mitochondrial genes with Mfannot [52] and these are available in electronic supplementary material, table S3.

As the genome completeness of the SAGs UC2 and UC3 was very low, we decided to continue the genome annotation only for the SAGs UC1 and UC4. We annotated the genome with AUGUSTUS [53] trained with CEGMA proteins [47], as explained in [34]. To predict the number of genes that may contain the full genome sequence of UC1 and UC4, we first performed a BLASTp v. 2.2.31+ [49] using as a query our predicted proteins against a database that includes all non-redundant proteins from UniProt [54] with a cut-off e-value of 1e-04, to identify the potential contaminant proteins. We removed the proteins that had the first hit from a bacterial or archaeal origin. However, as there were many genes without blast match and the annotation process can overpredict gene content [34], we decided to take into account only proteins in which, using a Pfam scan, we could find protein domains. The proteomes of *M. brevicollis* and *S. rosetta* contain approximately 70% of their proteins with a described Pfam protein domain. Therefore, we considered this fact in our calculations together with the average between the genomic completeness obtained by BUSCO and CEGMA (taking into account complete and fragmented proteins detected), to infer the total number of proteins that UC1 and UC4 may have in their complete genomes. SAGs assembly and annotation are available at Figshare (https://doi.org/10.6084/m9.figshare.7819571.v1), including the list of protein classification.

### (d) Genome size estimations UC1 and UC4

To estimate the genome sizes of UC1 and UC4, we took the average of the percentage of presence of BUSCO and CEGMA proteins (complete and fragmented), and the length of each assembly. Then, we inferred the putative genomic size of UC1 and UC4 if the percentage was 100%.

### (e) Ecological distribution of our SAGs

We performed a BLASTn v. 2.2.31 [49] using as query the 18S sequences of our SAGs against the operational taxonomic units (OTUs from the TARA Oceans database [55]. We found four OTUs that correspond to our SAGs with 100% or 99.2% identity (only one mismatch) (electronic supplementary material, table S4). We plotted the read distribution according to geographical locations using R [56].

### (f) 18S ribosomal gene phylogeny

We collected 18S rDNA ribosomal sequences from representatives of all known 18S rDNA molecular diversity available at public repositories of unicellular holozoans, including uncultured lineages Clade L [29], FRESCHOs, MACHOs and MAOPs [30] (electronic supplementary material, table S5). We ended up with a dataset of 117 18S rDNA sequences. Next, we aligned them using MAFFT [57] with the E-INS-i algorithm. After manual trimming sequence ends, indels and spuriously aligned sites we ended up with a total of 1754 sites, as we did in other 18S rDNA phylogenetic reconstructions [58,59]. As a control, we also aligned the sequences with MAFFT Q-INSI algorithm, which takes into account the structural RNA information [60] and the alignment was trimmed with trimAl (*automated1* argument) [61]. We inferred both phylogenetic trees from these alignments using a maximum-likelihood (ML) inference. The best substitution model for phylogenetic inference was selected using IQ-TREE [62], using the TESTNEW model selection procedure and following the Bayesian information criterion (BIC). In all four cases, the GTR substitution matrix with a 5-categories free-rate distribution [63] (a modification of the standard Γ distribution) was selected as the best-fitting model. ML inferences were performed with IQ-TREE, and statistical supports were drawn from 1000 ultrafast bootstrap values with a 0.99 minimum correlation as convergence criterion [64], and 1000 replicates of the SH-like approximate likelihood ratio test [65]. Both phylogenetic trees in nexus file and the alignments before and after the trimming are available at Figshare (https://doi.org/10.6084/m9.figshare.7819571.v1). Both trees show the same position of our SAGs, although the trimmed tree is more consistent with previous phylogenetic reconstructions of the 18S gene in choanoflagellates in which Craspedida appears monophyletic [27,30]. Thus, it is the one used to prepare the figure 1.

### (g) Eight-gene phylogeny

Similar to the recently published choanoflagellate phylogeny [27], we built a phylogenetic matrix with the nucleotide sequences of eight house-keeping genes, to infer the choanoflagellate phylogeny with a broader diversity than our phylogenomics approach. The genes used are: the ribosomal SSU (18S) and LSU (28S) genes, actin, beta tubulin, hsp90, hsp70, EF, and EF1A. Electronic supplementary material, table S6 summarizes the presence of each gene in each taxon. All the sequences used in the analysis are available at Figshare (https://figshare.com/s/9ed9c15e93bf4220868e). The analysis was performed with 66 taxa, 57 of them being choanoflagellates. To build the final matrix, we aligned each gene separately with MAFFT [57] using E-INS-i algorithm, and we next trimmed the spurious positions manually. Finally, we concatenated the trimmed alignments for each gene, building a phylogenetic matrix composed of 12 884 nucleotide positions. To run the phylogenetic analysis we partitioned our dataset into three parts, and in each of them we ran an evolutionary model with different rate distributions separating the ribosomal genes (partition 1), the 1st and 2nd codon positions of the non-ribosomal genes (partition 2), and the third codon position of the non-ribosomal genes (partition 3). The best substitution model for each partition was selected, again, using IQ-TREE [62], with the TESTNEW model selection procedure and following the BIC criterion. The ML analysis run with GTR substitution model with a 5-categories free-rate distribution [63] (a modification of the standard Γ distribution) was selected as the best-fitting model in the partition 1, with 3-categories in the partition 2 and with 4-categories in the partition 3. Statistical supports were drawn from 1000 ultrafast bootstrap values with a 0.99 minimum correlation as a convergence criterion [64]. Bayesian inference (BI) was performed with MrBayes 3.2.6 [66] using the GTR+ Γ model of nucleotide substitution in all partitions, running at different distributions according to the model given by IQ-TREE (Γ5, Γ3, Γ4 respectively for each partition). Four chains ran for 4 400 000 generations and converged (standard deviation of split frequencies = 0.02) and were analysed after a burn-in of 25%. The trimmed concatenated alignment, the partition information and the phylogenetic trees from ML and BI are available at Figshare (https://doi.org/10.6084/m9.figshare.7819571.v1).

### (h) Phylogenomic analysis of Amorphea using 87 single-copy protein domains and topological test

We updated the phylogenomic dataset developed in [13,14], consisting of 87 single-copy protein domains from 57 amorphean taxa, with our new data from SAGs UC1 and UC4. We also included the 19 new choanoflagellate transcriptomes [27,67], plus three species from the recently described holozoan genera *Pigoraptor* and *Syssomonas* [41]. We used a custom script [13] that uses tBLASTn alignments with an e-value cut-off of 0.05 [49] to search protein domains over the assembled genome. We recovered 32 and 20 protein domains for the SAGs UC1 and UC4, respectively, which accounted for 6844 and 6132 ungapped positions out of 22 201 ungapped positions of the consensus sequences of the final alignment. The final alignment contained 23 364 amino acid positions.

We built ML phylogenetic trees using IQ-TREE v. 1.5.1, under the LG model with a 7-categories free-rate distribution, and a frequency mixture model with 60 frequency component profiles based on CAT (LG+R7+C60) [64]. LG+R7 was selected as the best-fitting model according to the IQ-TREE TESTNEW algorithm and the BIC. The C60 CAT approximation was used to improve the rate of true topology inference [68]. Statistical support was obtained from 1000 ultrafast bootstrap values (correlation coefficient ≥ 0.99) [64] and 1000 replicates of the SH-like approximate likelihood ratio test (electronic supplementary material, figure S1) [65].

The same alignment was used to build a Bayesian inference tree with Phylobayes MPI 755 v. 1.5, using the LG exchange rate matrix with a 7-categories gamma distribution and the non-parametric CAT model (LG+Γ7+CAT) [69], removing constant sites to reduce computation time. We used a Γ7 distribution instead of a R7 distribution (as suggested for the IQ-TREE ML analysis) because free-rates distributions are not implemented in Phylobayes. We used two independent chains that were run for 5660 and 5685 generations, respectively, until convergence was achieved (maximum discrepancy = 0.0851376) with a burn-in value of 13% (739 burnt-in trees). The adequate burn-in value was selected by sequentially increasing the number of burn-in trees, until (i) the maximum discrepancy statistic reached the threshold of less than 0.01 and (ii) we maximized the effect size of the log-likelihood parameter. The sampled trees had a maximum discrepancy = 0.0851376, a mean discrepancy = 0.00130004 (as per the bpcomp analysis in Phylobayes) and a minimum effective size for the log-likelihood parameter = 4 (tracecomp analysis). The trimmed alignment and the phylogenetic trees from ML and BI analysis are available at Figshare (https://doi.org/10.6084/m9.figshare.7819571.v1). Finally, the topology test was performed with IQ-TREE v. 1.5.1 under the LG+R7+C60 model.

### (i) Comparative genomics by protein domain gains and loses

116 different eukaryotic taxa with proteomic information available were selected to perform an analysis of protein gains and losses over the eukaryotic tree of life focusing on holozoans (56 taxa) (electronic supplementary material, table S7, and supplementary information at Figshare https://doi.org/10.6084/m9.figshare.7819571.v1). Protein domain annotations of each proteome were computed using Pfamscan and the 29th release of the Pfam database [70]. We used a custom script to build a matrix containing the eukaryotic taxa and the number of copies of each protein domain. To reduce noise and eliminate possible contaminants, we removed all the protein domains that were found in 95% (or more) of cases within prokaryotic species (Bacteria and Archaea) according to Pfam database. We ended up with a matrix of 116 taxa and 8920 protein domains. Next, we produced a tree nexus file according to the topology of eukaryotes [71]. For unicellular holozoans we incorporated the topology of our phylogenomic analysis. With the protein domain matrix and the consensus taxa tree we used Count [72] to infer the gains and losses for each node of the tree using Dollo parsimony. Using Count, the domains gained at the different ancestral nodes of holozoans could be retrieved. The functional annotation of the 120 protein domains gains at Choanozoa was done manually by checking the literature available for each protein domain. The list of different protein domains across Opisthokonta ancestors (Opisthokonta, Holozoa, Filozoa, Choanozoa, and Metazoa) is available at Figshare (https://doi.org/10.6084/m9.figshare.7819571.v1) together with the protein domain matrix used.

### (j) The probability of retention in extant species

With the list of proteins domains gained at the ancestral nodes, we calculated the probability of conservation of a given protein domain in a phylogenetic group by dividing the number of species of this group (i.e. animals) that have maintained this protein domain by the total number of species of this group (i.e. all the animals present in our analysis). The list of probabilities of retention is joined to the list of proteins domains gained at the different ancestral nodes within Figshare (https://doi.org/10.6084/m9.figshare.7819571.v1). The distribution of these probabilities has been plotted in R [56]. SAGs UC1 and UC4 were not included in this probabilistic analysis given that their fragmented genomes will underestimate the probability of retention in choanoflagellates.

### (k) Pou, Plexin and Nucleophosmin phylogenies

We studied the phylogenetic history of Pou transcription factor, Plexin proteins and the C-terminal domain of Nucleophosmin in detail by following similar approaches. We selected the proteins or the protein domains present in choanoflagellate species and also in a set of metazoan species in which most of animal phyla are represented (electronic supplementary material, table S7). We aligned the sequences using MAFFT [60] and trimmed the alignment with trimAl [61]. The phylogenetic inferences were done with IQ-TREE v. 1.5.1, under the best-fitting LG model. The alignments and the trees can be found in the electronic supplementary material (https://doi.org/10.6084/m9.figshare.7819571.v1).

## 3. Results

### (a) Expanding the genomic diversity of choanoflagellates

To broaden our understanding of the genomic diversity among choanoflagellates, we sequenced four single-cell amplified genomes corresponding to uncultured choanoflagellate cells collected during the TARA oceans expedition [55] (see electronic supplementary material, table S1 for collection environmental details). The four cells belonged to different choanoflagellate taxa, and they did not appear to be related to any previously described species with transcriptomic or genomic information available (figure 1).

To place the different SAGs within the choanoflagellate tree, we first performed a phylogeny of the 18S ribosomal subunit that included the SAGs and the known 18S molecular diversity of unicellular holozoans, including environmental sequences [30]. UC1 appears as an early-branching clade 1 craspedidan that groups with *Lagenoeca antarctica* [73] (figure 1). Its 18S sequence is identical to the environmental NCBI sequence AY426842 (100% of pairwise identity). UC2 forms a monophyletic clade with the rest of Acanthoecidae (nudiform loricates) (figure 1) appearing as sister-group to the rest of acanthoecids. UC3 clusters with the tectiform loricates *Stephanoeca paucicostata* and *Stephanoeca cauliculata*. Finally, UC4 falls as the earliest-branching sister to Acanthoecida, together with the environmental sequence JQ223245. Thus, the four cells belonged to different choanoflagellate taxa and were not related to any previously described species (figure 1). Moreover, they appeared distantly related to the two choanoflagellates species with a whole genome sequence (*Monosiga brevicollis* and *Salpingoeca rosetta*), thus expanding the genomic information currently available for choanoflagellates.

We then analysed the geographical distribution of these uncultured choanoflagellates using the metabarcoding data from the TARA Oceans database [55]. We found that the craspedidan UC1 is mainly present in Mediterranean samples, although not exclusively (electronic supplementary material, figure S2A). Interestingly, the environmental sequence AY426842 (which is identical to UC1) was also sampled in the Mediterranean [74]. Acanthoecida sister UC4 is the third most abundant choanoflagellate in TARA Oceans, and it has a cosmopolitan distribution (present in 46 sampling stations out of 47) (electronic supplementary material, figure S2A). The nudiform UC2 and the tectiform UC3 are also widely distributed (45 samples out of 47), albeit less abundant than UC4 (electronic supplementary material, figure S2B). Since most of the TARA Oceans reads associated to our SAGs appear in the picoplanktonic fraction (electronic supplementary material, figure S2B) our SAGs' cell size likely ranges between 0.8 and 5 µm, in agreement with the typical size range of described choanoflagellate species [25]. Furthermore, our four SAGs are relatively more abundant in surface waters than in deeper sampling points such as the deep chlorophyl maximum (electronic supplementary material, figure S2B).

### (b) Genome completeness and statistics of the SAGs

Once we had deciphered the taxonomy and the ecological distributions of our SAGs, we sequenced their genomes using Illumina Miseq as in [34] (see §2 for further details). We then checked the genome recovery and the genome statistics of our final assemblies, including the estimation of genome completeness by BUSCO [48]. UC1 and UC4 presented a significant genome recovery (7.74 MB and 31.68% BUSCO for UC1; 7.25 Mb and 13.53% of BUSCO for UC4) (table 1; electronic supplementary material, table S2). However, UC2 and UC3 were mostly incomplete and fragmented (table 1), and for this reason they were not included in most of the subsequent analyses, except for the eight-gene based phylogeny (electronic supplementary material, figure S3). Interestingly, we were able to recover the mitochondrial genome of UC2 (table 1), which is the first available mitochondrial genome of an acanthoecid choanoflagellate. We could annotate 59 mitochondrial genes (electronic supplementary material, table S3), which revealed a high degree of conservation with the mitochondrial genome of *M. brevicollis* [75].

**Table 1.** Summary of the genome statistics of each SAG assembly.

SAG	taxonomy	scaffolds^a^	largest scaffold (bp)	N50	total length (Mb)	GC (%)	CEGMA (%)	Busco (%)
UC1	Craspedida clade 1	3276	41 637	4928	7.74	49.8	20.1	31.7
UC2	Acanthoecidae	746	32 186	1499	1.00	30.8	0.8	0.7
UC3	Stephanocidae	819	11 187	2197	1.31	33.5	—	0.3
UC4	Basal Acanthoecida	2527	72 672	11360	7.25	40.0	14.1	13.5

^a^Scaffolds bigger than 500 bp.

In order to extrapolate the putative genome size of our SAGs, we performed an estimation using BUSCO end CEGMA values (see §2). The results showed that the craspedidan UC1 (29.4 Mb, see table 2) would potentially contain the smallest genome among the so far sequenced choanoflagellates; *S. rosetta* (55.4 Mb) and *M. brevicollis* (41.6 Mb). The predicted genome length of the early-branching UC4 (52.5 Mb) is similar to that of *S. rosetta*. However, it is worth mentioning that this approach can yield biased results, because it assumes that core eukaryotic genes are homogeneously distributed along the genome, it does not differentially account for fragmented and complete genes, and it does not account for the lower detection rate of fragmented genes inherent to all gene prediction algorithms [53]. Thus, the results have to be interpreted cautiously. Using this approach with previously published SAG of the choanoflagellate *M. brevicollis* [34] with a BUSCO values above 12% (similar to UC1 and UC4) results in an approximately 10% over-estimation of its genome size relative to the reference genome [9]. Finally, it is worth mentioning that alternative methods of genome size estimation, such as *k*-mer frequency distribution, are unfortunately not adequate for SAG assemblies, as they require that sequencing coverage along the genome be unbiased [76], a condition not fulfilled by SAG-derived short reads [34]. Altogether, these observations suggest that genome size estimates in SAG data suffer from inherent biases and have to be interpreted with caution.

**Table 2.** Genome estimation of our SAGs^†^ within choanoflagellate context.

genome	assembly size (Mb)	genome size (Mb)	no. of annotated genes	total no. of genes
UC1	7.74	29.4^†^	3025	6039^†^
UC4	7.25	52.5^†^	2518	10 075^†^
*Salpingoeca rosetta*	—	55.4	—	11 624
*Monosiga brevicollis*	—	41.6	—	9172

Next, we predicted the number of genes that might contain the full genomic sequences of UC1 and UC4 taxa, by extrapolating the numbers of genes annotated with the BUSCO/CEGMA values, removing the potential contamination and taking into account Pfam protein domain predictions (see §2). The difference in estimated size is proportional to the number of estimated genes. UC1 has a smaller number of genes (6039) according to the predicted reduced genome size, and UC4 would present a more similar number of genes than the previous choanoflagellate genomes (10 075) (table 2).

We then characterized our SAGs by screening for genes linked to morphological structures, such as the microvilli or the lorica of acanthoecids, in order to speculate on the potential morphology of these two choanoflagellate taxa. Therefore, we searched for the presence of Ezrin/Radixin/Moesin (ERM) protein [77,78], which is known to be involved in microvilli elongation processes; as well as for the presence of Si transporters (SITs) [79], needed for the lorica formation in Acanthoecida. The microvilli-related ERM protein was only found in the UC4 genome, and not detected in UC1. We also failed to identify any SIT in any of those taxa, including the sister Achantoecida UC4 [79]. Thus, with these results and without complete genomic data we cannot speculate whether these choanoflagellates present Lorica or not.

### (c) Phylogenomics confidently reconstruct the phylogenetic position of UC4 and UC1 and raise questions regarding deep phylogenetic branches of choanoflagellates

**Figure 2.**
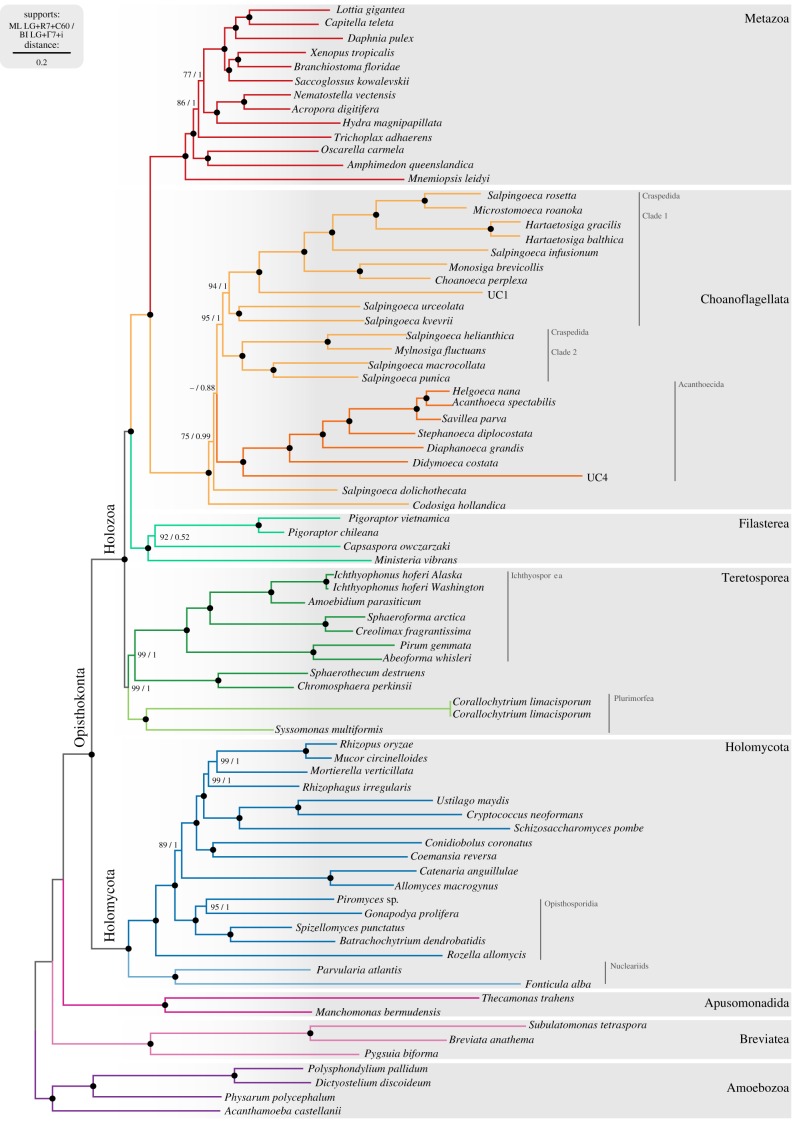
Phylogenomic tree of holozoans. Phylogenomic analysis of 87 single-copy protein domains [14] accounting for 23 364 amino acid positions. Tree topology is the consensus of two Markov chain Monte Carlo chains run for 5660 and 5685 generations, after a burn-in of 13%. Statistical supports are indicated at each node: on the left, non-parametric ML ultrafast-bootstrap (UFBS) values obtained from 1000 replicates using IQ-TREE and the LG+R7+C60 model; on the right, Bayesian posterior probabilities (BPP) under the LG+Γ7+CAT model as implemented in Phylobayes. Nodes with maximum support values (BPP = 1 and UFBS = 100) are indicated with a black bullet. Raw trees are available on Figshare (https://doi.org/10.6084/m9.figshare.7819571.v1) and electronic supplementary material, figure S1 shows the topology and the supports of the ML inference.

To reconstruct the evolutionary history of the different protein domains from the LECA to extant animals, we need a proper phylogenetic framework. Moreover, we were also interested in confidently placing our SAGs within the phylogeny of choanoflagellates. We, thus, built a phylogenomic matrix based on 87 single-copy protein domains [14] over 79 taxa including animals and all their unicellular relatives with available transcriptomic or genomic data. We therefore included the SAGs UC1 and UC4, the recent transcriptomic data of 19 choanoflagellates [23] plus the two choanoflagellates with complete genome sequences, *M. brevicollis* and *S. rosetta* [9,10]. In addition, we included the four known filasterean taxa, including the recently described genera *Pigoraptor*. We also included all ichthyosporean taxa with genome information, the early branching ichthyosporean *Chromospharea* *perkinsii*, as well as the plurimorfean (or corallochotryeans) Corallochytrium and *Syssomonas* [14,41] (figure 2). Finally, we included an extensive outgroup composed by holomycotans (18 taxa), apusomonads (2 taxa), breviates (3 taxa) and amoebozoans (4 taxa).

Our tree recovers monophyly of choanoflagellates with maximum support (figure 2). Our SAGs UC1 and UC4 were confidently placed within choanoflagellates. UC4 is confirmed to be an early-branching sister to Acanthoecida. Thus, UC4 falls in a key phylogenetic position to better understand Choanoflagellatea and Acanthoecida evolution. Given its high abundance, it might also be an important ecological player. On the other hand, UC1 is confirmed to be a clade 1 craspedidan, as in the 18S rRNA tree (figure 1), appearing as sister-group to the previously described craspedidan clade 1 [27].

However, and somehow unexpectedly, our tree recovered some important topological differences compared to previous choanoflagellate phylogenies based on a few genes [24,27]. The main one is that in our tree Craspedida appears paraphyletic (figure 2). Interestingly, *Codosiga hollandica* appears as sister-group to the rest of the choanoflagellates prior to the split of *Salpingoeca dolicothecata* and the divergence of Craspedida and Acanthoecida. This position for *C. hollandica* is relatively well supported (75% ML UFBS/0.99pp BI), although the nodal supports increase a bit when the fastest 2500 evolving sites are removed (electronic supplementary material, figure S4); however, nodal support decreases again if more positions are removed from the alignment (electronic supplementary material, figure S4). Hence, our extended analyses show that some relationships are unstable. For instance, the next branching after *C. hollandica*, in our Bayesian reconstruction is *S. dolicothecata* prior to the divergence of Craspedida and Achanthoecida plus UC4, but in the ML inference, *S*. *dolicothecata* falls sister to Acanthaoecida and UC4 (figure 2 and electronic supplementary material, figure S1). Moreover, different topological tests were not able to discard any of the following alternative hypotheses: (1) *C. hollandica* as the earliest branching lineage and *S. dolicothecata* sister to the clade formed by the rest of craspedidans and Acanthoecida; (2) *S. dolicothecata* branching as sister to Acanthoecida and UC4 clade and *C. hollandica* remaining early branching; and (3) the classical view in which Craspedida and Acanthoecida are monophyletic (electronic supplementary material, figure S5). In addition, our eight-gene based phylogeny using a broader taxon sampling, similar to the most recent, and taxon-rich, phylogenetic reconstruction of choanoflagellates [27], brings *C. hollandica* together with other codosiga species within clade 2 of Craspedida. But still, before the split Acanthoecida–Craspedida there is the craspedidan clade 3 composed by *S. dolicothecata* and *Salpingoeca tuba* appearing as the earliest branching clade (electronic supplementary material, figure S3).

Thus, our data suggest that with the current information we cannot properly tackle deep choanoflagellate relationships. We need more genomic information from broader taxon sampling, as well as further understanding of choanoflagellate diversity. Previously described environmental clades like Clade L [29] or FRESCHO3-4 groups [30] that branch in early positions in the 18S rRNA phylogeny (figure 1), together with the species *S. tuba* and others from the genera *Codosiga* and *Sphaeroeca* (related to *Codosiga* in the 18S rRNA (figure 1) and in the eight-gene phylogenies; electronic supplementary material, figure S3) will help to solve these deep choanoflagellate relationships by increasing our knowledge of choanoflagellate diversity in missing (and key) phylogenetic positions.

Another interesting result from our phylogenetic reconstruction is that *Salpingoeca urceolata* and *Salpingoeca kvevrii* appear as sister-group to clade 1 and not within clade 2, as previously described in [27]. Therefore, our results might redefine these two groups of craspedidans (figure 2). On the other hand, our data show with high nodal support that nudiforms cluster within tectiforms, meaning that nudiforms and tectiforms are not two independent lineages within Acanthoecida.

Finally, our results recovered, within unicellular holozoans, the monophyly of Teretosporea [13] with high nodal support (99% of ultra-fast bootstrap from ML (UFBS) and 1 of posterior probability of Bayesian inference (BI) (figure 2)). Apparently, the addition of *Syssomonas* and *Chromosphaera* together in the same phylogeny allows better statistical support than obtained in previous studies [14,41]. Therefore, this is the phylogenetic framework we used in our subsequent protein domain evolution reconstruction analysis.

### (d) The Urmetazoan genome did not experience an increase of innovation at the level of protein domains

**Figure 3.**
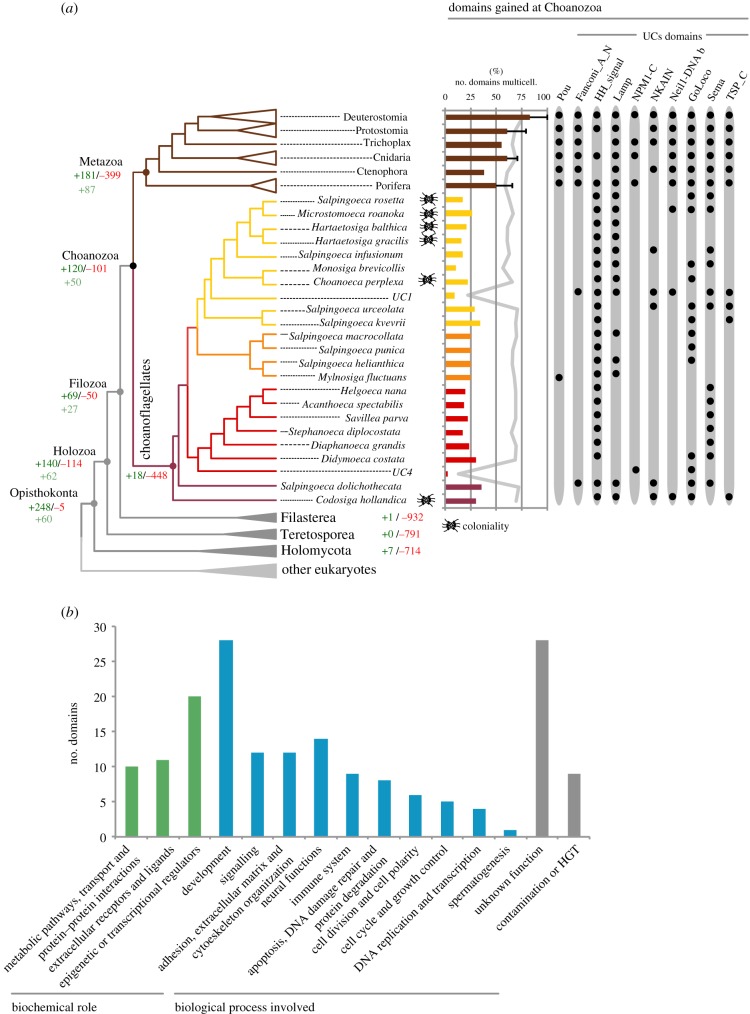
Summary of proteins gains and losses in Opisthokonta, focusing on Choanozoa gains. (*a*) Schematic of the choanoflagellate phylogeny obtained, including the numbers of protein domains gains and losses in each Opisthokonta clade (depicted in green and red respectively). Light green numbers represent the protein domain gains that have retention of over 70% in extant metazoan species, in a given ancestor. Protein domains from potential bacterial or archeal contamination were excluded from the analysis (see §2). The ability to form colonies (marked with a colony drawing) is shown on the right, and has been adapted from [27]. Our SAGs (UC1 and UC4) are marked in italic. Next to the tree, there is a bar chart indicating the percentage of protein domains gained at Choanozoa and judged to be involved in animal multicellular processes (a total of 69 domains out of 120), retained in each choanoflagellate taxa. Animal data are displayed by phylum instead of species, thus what it is shown is the average and the distribution of domains kept by all the analyzed species of each animal phylum. As a control, the retention of all protein domains that have the Choanozoan ancestor among extant species is shown in grey. Further to the right, the POU protein domain distribution and the protein domains gained at Choanozoa are shown, which are present in our sequenced SAGs UC1 and UC4. A black dot indicates the presence of each domain in the different taxa/clade. (*b*) Function of the protein domains gained at Choanozoa. In green, the biochemical roles in which the protein domains are involved. In blue, the biological processes in which the domains have been shown to participate. These two classifications are not exclusive; one protein domain can appear in one or multiple categories. In grey, protein domains with unknown function, or contaminants or a product of an horizontal gene transfer (HGT) event.

After establishing the taxonomical framework, we analysed the evolutionary history of protein domains from LECA to animals. To perform this analysis, we built a database of 116 proteomes of different eukaryotic taxa (electronic supplementary material, table S7), representing the entire eukaryotic diversity. We then predicted the protein domain architectures and produced a matrix of presence/absence of each protein domain across all the eukaryotic taxa (see §2). Finally, we inferred the gains and losses among each node of the eukaryotic tree of life by Dollo parsimony. The results shown are focused on opisthokonts (figure 3), using the taxonomical framework obtained in our phylogenomic analysis; except for Ctenophora, in which we assumed Porifera as the earliest branching animal lineage. This phylogenetic framework is in accordance with recent phylogenomic analyses [67] and previous studies of animal genomic reconstructions [14,22,23] and it reduces the effect of the excess of gene losses in the fast-evolving ctenophore *Mnemiopsis leydi* [22].

The first observation is that the number of novel protein domains gained in the last common ancestor (LCA) of Metazoa (181) is in line with the number of gains in previous LCAs since the origin of Opisthokonta (domains gained: 248 Opisthokonta LCA, 140 Holozoa LCA, 69 Filozoa LCA, 120 Choanozoa LCA; figure 3), without an important increase of new protein domain acquisitions at the stem of Metazoa compared with its unicellular ancestors. On the other hand, these gains are offset by a high number of protein domain losses compared with the Choanozoan ancestor (animals+choanoflagellates) (from 5715 to 5497 protein domains).

Among the losses at the stem of metazoans, we found protein domains involved in the biosynthesis of essential amino acids (for instance: Anth_synt_I_N, Shikimate_dh_N, PDT) and carbohydrate metabolism (Pantoate_ligase, Glyco_transf_34) as previously suggested [23,80–82].

In addition, we observed an important fraction of protein domains lost that had a bacterial ancestry (i.e. ion transporters; see supplementary material at Figshare https://doi.org/10.6084/m9.figshare.7819571.v1). Thus, with our analysis it is difficult to disentangle which is the real origin of these protein domains. We can expect that most of the domains were already present in the LECA inherited from vertical transmission from prokaryotic ancestors, but others might have been acquired in extant eukaryotic lineages by horizontal gene transfer; for example, bacterial rhodopsins (domain Bac_rhodopsin) are described as an horizontal gene transfer from bacteria to eukaryotes [83]. We discard the possibility of these domains coming from contaminants because the genomes/transcriptomes used in our dataset are of good quality and we were very strict in the treatment of possible contaminations in our SAGs data (see §2).

Thus, in order to have more data to interpret these results, we compared the retention of these Metazoan-lost protein domains with the retention of all protein domains present at the LECA, in the rest of eukaryotic species that were not animals. The results show that the domains lost in Metazoa are less retained in the rest of eukaryotic species than all protein domains present at LECA (a median of 18% of retention for metazoan lost domains compared with a 60% of retention of all domains present at LECA; electronic supplementary material, figure S6). Actually, only 40 out of the 399 protein domains lost at Metazoa are retained in half or more of the rest of eukaryotic species (electronic supplementary material, table S8). Among these 40 clear Metazoan losses are included the protein domains mentioned above that are related in amino acid biosynthesis and carbohydrate metabolism (electronic supplementary material, table S8).

Thus, either most of these protein domains lost in metazoans were vertically acquired from the LECA to the Choanozoan ancestor and subsequentially lost in animals, which will imply major losses in other eukaryotic lineages, or most of these protein domains are recent acquisitions by horizontal gene transfer events from bacteria to some extant eukaryotic lineages, including Choanoflagellates. Probably both scenarios are occurring in our metazoan losses, but without a detailed phylogenetic analysis of these domains we cannot discover to what extent horizontal gene transfer events are affecting our results.

Thus overall, our data show that, at the stem of animals, there was not an important increase in new protein domains, and at the same time, many important metabolic functions were lost. Therefore, if we compare our results with previous studies that had shown an important acquisition of new genes in the origins of animals [14,22,23]—and that some of these acquisitions were the product of domain shuffling events [9,10,12,14]—we might conclude that such appearance of new gene families at the onset of Metazoa was facilitated by a massive rearrangement and duplication of pre-existent protein domains and not by the gain of new protein domains.

Finally, it is worth mentioning that an important fraction of the losses observed might correspond to horizontal gene transfer events to the unicellular relatives of animals, thus it might be the case that there was not a net loss of protein domains at the stem of Metazoa.

### (e) Pre-metazoan protein domains essential for animal multicellularity are not retained in their unicellular relatives

**Figure 4.**
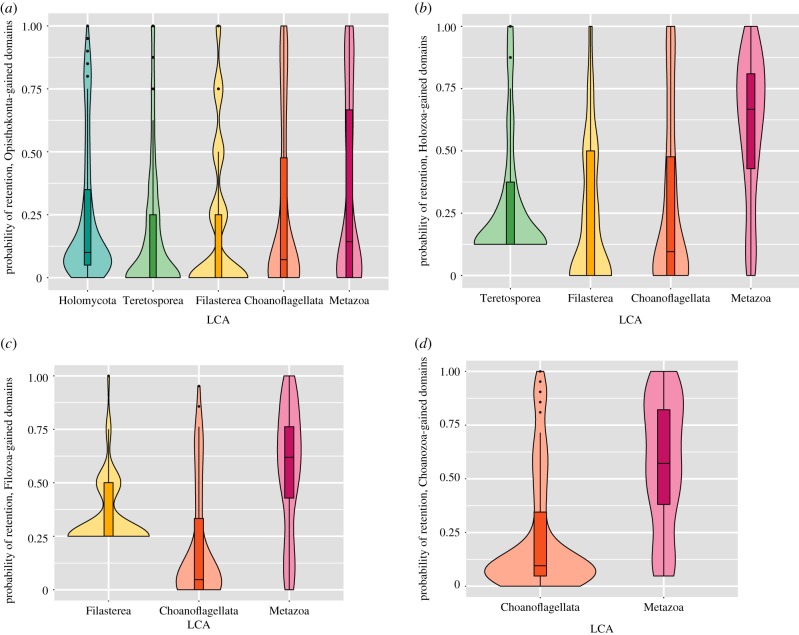
Distribution of the probability of retention of the protein domains acquired in the different ancestors: Opisthokonta (*a*), Holozoa (*b*), Filozoa (*c*) and Choanozoa (*d*) in the extant species.

Next, we questioned which of these pre-existing protein domains were more important in the transition towards animal multicellularity, and when they appeared. To analyse this, for each domain acquired in the successive ancestors from the last Opisthokonta common ancestor (LOCA) to the Urmetazoa, we plotted the probability of retention on extant metazoan species and their unicellular relatives (figure 4). SAGs UC1 and UC4 were not taken into account for this probabilistic analysis because their genomes are not complete, and therefore the lack of data would underestimate the probability of protein domain retention in choanoflagellates. The rest of the eukaryotic species used in the analysis have high BUSCO values (greater than 90% in most cases) and present similar numbers of total protein domains (mostly between 3000 and 4500; electronic supplementary material, table S9), except for vertebrate species (3 out of 21 Metazoans), which are better studied and present around 5500 protein domains, and the parasitic species of Microsporidia (around 1000). Since we have 22 other holomycotan species, we do not consider that the low number of genes in Microsporidia would significantly affect our results (electronic supplementary material, table S9).

The results show that the protein domains acquired at the base of opisthokonts were proportionally less retained in the extant metazoan species than the protein domains acquired in the successive Holozoan ancestors. This implies that an important fraction of the 248 protein domains gains occurred at LOCA that are not crucial for animal functions. On the other hand, the protein domains gained at the stem of Holozoa and the successive unicellular ancestors (Filozoa and Choanozoa) are likely to be retained in extant animal species, but not in their unicellular relatives (figure 4). Figure 3*a* shows the number of protein domain gains that have retention of over 70% in extant metazoan species. We can observe that unicellular holozoan ancestors contributed significantly in the acquisition of these conserved protein domains (139 in total; 62 Holozoa, 27 Filozoa, and 50 Choanozoa). Finally, protein domains acquired at the origins of animals have the highest rates of retention in extant animals (87 protein domain innovations were retained by more than 70% of metazoan taxa).

Therefore, the protein domains acquired at the origins of animals seems to be more essential for animal functions because they tend to be more conserved. However, proportionally, the protein domains gained in the different unicellular holozoan ancestors are also key for maintaining such functions, presenting a high degree of conservation among extant animal species compared with their unicellular counterparts.

### (f) Key protein domains for animal multicellularity

To get a better understanding of the set of conserved protein domains from LOCA to the Urmetazoa, we list in table 3 the protein domains that were retained in all animal species here analysed (21 species). Those protein domains are related to biological processes that have been mainly hypothesized to be required for the evolution of animal multicellularity. These processes include gene expression regulation, cell-to-cell communications and cell adhesion [9,12,20,84,85]. Most of the domains listed predate the origins of animal multicellularity. Protein domains like T-Box, runt, Integrin_beta_2 and Laminin_N were already described in proteins shown to be of pre-metazoan origin [16,19,85,86]. However, our analysis also shows highly conserved protein domains that predate the origins of animals, like LEM or NUDE_C. LEM acts in a protein of the inner nuclear membrane involved in the chromatin organization and the post-mitotic re-assembly of the nucleus [87]. NUDE_C is the C terminal protein domain of the NDE1 protein, which is required for centrosome duplications and formation and the correct functioning of the mitotic spindle. It has also been described as essential for the development of the cerebral cortex, by controlling the orientation of the mitotic spindle in cortical neuronal progenitors [88]. Thus, besides cell-to-cell communications, cell adhesion and gene expression regulation, our results suggest that other more general eukaryotic functions, such as chromatin organization, cell division, ubiquitination (beta-TrCP_D domain) or translational regulation at ribosomal scale (RAC_head domain) (table 3) were more specialized in metazoans mediated by these highly conserved proteinic domains. Finally, we also detected among these ‘essential domains’ that predate the origins of animals, protein domains of unknown function, such as Calpolin (table 3).

**Table 3.** Summary of the protein domains acquired before and at the origins of animals, which are maintained by all 21 metazoan extant species used in this analysis.

origin	protein domains retained	protein domain information
Opisthokonta	transcription factors and DNA binding domains	
	*T-box*	transcription factor involved in animal development
	*BTD*	nuclear effector of Notch signalling
	*LAG1-DNAbind*	related to BTD, nuclear effector of Notch signalling
	*NUDE_C*	involved in chromosome migration
	*PAS_11*	interacts with STAT6 transcription factor
	signalling and GTPase interactors	
	*Arfaptin*	involved in the vesicle budding at Golgi apparatus
	*GIT_SHD*	signalling integrators with GTPase activity
	translational regulator	
	*RAC_head*	involved in ribosomal binding
	unknown function	
	*Calpolin*	
	*HS1_rep*	
	*DUF3518*	
	*DUF3585*	
Holozoa	signalling binding-related domains	
	*GKAP*	interacts with guanylate kinase-like domain
	*PID*	phosphotyrosine interacting domain
	*LLGL*	known to be present in syntaxin-binding proteins
	nuclear membrane protein	
	*LEM*	found in inner nuclear membranes
	transcription factor	
	*Runt*	transcription factor related to animal development
	unknown function	
	*DUF1908*	
Filozoa	signalling and adhesion	
	*Integrin_alpha2*	extracellular domain of integrins
	ubiquitination	
	*Beta-TrCP_D*	D domain of beta-TrCP that acts as ubiquitin ligase
Choanozoa	transcription factors	
	*MH1*	DNA binding domain of Smad TF
	*MH2*	domain that interacts with Smad TF regulators
	*Pou*	domain related with Homeobox superfamily
	extracellular matrix protein domains	
	*Laminin_N*	N terminal domain of laminins, extracellular proteins related to cell adhesion
	*P4Ha_N*	domain from prolyl 4-hydroxylase that is important in the post-translational modification of collagen
	*TSP_C*	C terminal domain of Thrombospondin, an adhesive glycoprotein that mediates cell-to-cell and cell-to-ECM interactions
	protein–protein interactions	
	*PET*	suggested to be involved in protein–protein interactions
	lysosomal protein	
	*Lamp*	integral membrane proteins of the lysosome with unclear functions
Metazoa	signalling	
	*wnt*	Wnt signal transduction pathways
	*TGF_beta*	transforming growth factor beta, regulatory peptides that generate intracellular signals
	*PP2C_C*	C terminal of serine/threonine phosphatase, the domain may provide specificity to the reaction
	transcription factors	
	*Ets*	transcription factor involved in multiple processes, cell differentiation, migration, etc.
	*Hormone_receptor*	ligand-binding domain of nuclear receptors that sense steroid and thyroid hormones
	extracellular matrix protease	
	*ADAM_CR*	membrane-anchored protease that modifies the ECM
	protein–protein interaction	
	*Death*	interaction protein module. Related with death effector domain and caspase recruitment domain

On the other hand, we found seven protein domains that were gained at the origin of animals and conserved in all of the 21 metazoan species analysed. Most of these protein domains have already been described as animal-specific, like two components of major signalling pathways (Wnt and TGF-beta); and two essential transcription factors families, Ets and nuclear hormone receptors (hormone_receptor) [20,22,23,85]. However, we also detected protein domains that are known to modulate the activity of different proteins by changing the specificity among protein–protein interactions, like the C-terminal domain of serine/threonine phosphatase (PP2C_C) and the Death domain.

### (g) Transcription factor innovations at Choanozoa: Pou TF predates animal origins

Thanks to our single-cell amplified genomes from the choanoflagellates UC1 and UC4, and to our protein domain reconstruction analysis, we have now a more comprehensive view of the genetic content of the Choanozoan ancestor. Our analysis revealed that 120 domains were gained at the stem of Choanozoans and we were interested in understanding the biochemical roles or biological processes in which those domains are involved. The results are depicted in figure 3*b* and show that most of the protein domains with known biochemical roles belong to transcription factors or epigenetic regulators. These include the protein domains POU and zf-C4, both previously thought to be animal-specific [22,85]. POU is the N-terminal protein domain of the POU homeobox gene family. POU genes are known for their roles in cell-type specification and developmental regulation in animals [89]; hence they are essential genes for animal multicellularity (table 3). Among choanoflagellates, we only identified the POU protein domain in the choanoflagellate species *Mylnosiga fluctuans*, appearing together with a homeobox domain, adopting the animal-like structure of POU genes.

**Figure 5.**
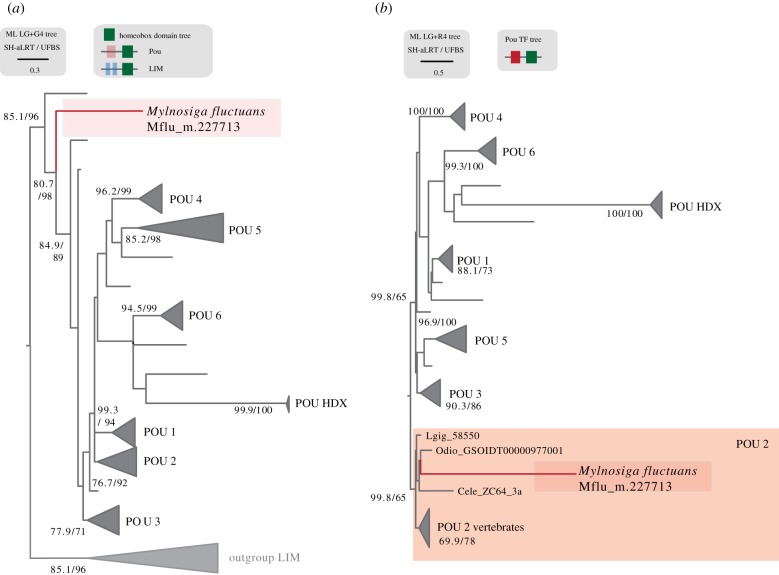
POU phylogenetic tree. (*a*) ML inference of Homeobox domain using LIM homeobox as an outgroup. LIM domains were downloaded from (http://homeodb.zoo.ox.ac.uk/download.get). Pou domains were selected from a wide range of metazoans available in our dataset, plus the choanoflagellate sequence (marked in red). Supports are SH-like approximate likelihood ratio test (left) and UFBS, respectively (right) calculated with IQ-TREE v. 1.5.1. (*b*) Maximum-likelihood inference of Pou transcription factors using the whole protein, the Pou domain and the Homebox domain. *M. fluctuans* Pou sequence falls within POU-2 group (marked in red).

In order to confirm that POU homeobox transcription factors were already present in the Choanozoa ancestor, we performed a phylogenetic analysis of the adjacent homeobox domain (which is conserved at the pan-eukaryotic level [90]), using LIM-associated homeobox as outgroup [90] (figure 5). The phylogenetic reconstruction places *Mylnosiga*'s homeobox domain within the animal POU family with high nodal support (98% UFBS; figure 5*a*). It falls in an early-branching position, before the expansion into different paralogs in animal species [89]. Thus, according to the results, we consider *Mylnosiga*'s POU homeobox domain as a *bona fide* POU orthologue. While sequence contamination could potentially explain the presence of this POU homeobox in *Mylnosiga*, two observations render this possibility unlikely: first, POU domains were previously considered to be animal-specific, thus ruling out non-animal contaminants [86]; second, the peptide sequence of the homeobox domain is clearly different from canonical animal POUs and consistent with the phylogenetic relationships between choanoflagellates and animals (early-branching; figure 5*a*). Furthermore, secondary losses of POU N-terminal domains have already been reported within *bona fide* animal homologues of the POU homeobox class [90], which can explain the low level of conservation of the associated POU domain in *Mlynosiga* (see alignment in supplementary material, Figshare https://doi.org/10.6084/m9.figshare.7819571.v1).

Finally, in order to clarify the POU phylogeny, we performed a joint phylogenetic analysis of the POU and homeobox domains. This analysis of the whole protein (POU plus homeobox domain, figure 5*b*) revealed a moderate improvement in the nodal support in most POU classes nodes. Surprisingly, *Mylnosiga* POU protein was associated to POU class 2 proteins, although with low bootstrap support (65% UFBS; figure 5*b*). Given that POU class 2 is reported to have appeared at the stem of Bilateria [86], this scenario would imply a rampant gene loss of Pou classes in non-bilaterian animals and in choanoflagellates. This scenario is not very parsimonious, and the association of *Mylnosiga* to POU class 2 proteins might also be explained as an artefact owing to the lack of resolution in our phylogenetic inference. More genomic sequences of non-bilaterian and choanoflagellate organisms could help resolve this issue.

On the other hand, we also detected the zf-C4 domain in choanoflagellate species. The zf-C4 domain is the DNA binding domain of the nuclear hormone receptors. We identified the domain alone in choanoflagellate protein sequences, without the animal-like structure, in which the zf-C4 domain is accompanied by the hormone_receptor Pfam domain.

The hormone_receptor domain is animal-specific and highly conserved in extant metazoans (table 3), having been lost only in the ctenophore *M. leydi* and in the poriferan *Oscarella carmela.* Thus, nuclear hormone receptors remain animal innovations under the current taxon sampling, but the DNA binding domain zf-C4 predates the origin of animals.

Finally, we also detected the presence of two protein domains, MH1 and MH2, that are highly conserved among metazoans (table 3) and were recently found as Choanozoan innovations [23]. MH1 is the DNA binding protein of Smad transcription factors that together with MH2 form the canonical Smad proteins [91]. Both MH1 and MH2 have a Choanozoan origin even though the canonical Smad architecture remains metazoan-specific, as previously described [20,85]. Thus, Smad proteins might be the result of a domain shuffling event at the stem of Metazoa, as already described [23].

### (h) Colonial choanoflagellates do not specially retain protein domains related in multicellular-like functions

We identified that protein domains that appeared at the origin of animals and choanoflagellates are involved in crucial functions to maintain animal multicellularity (figure 4*b*). These are protein domains involved not only in development, cell-to-cell signalling or adhesion, but also in other multicellular functions [19] such as neural functions, immunologic response [9,10,23], cell cycle control, or the control of cell polarity and division. Those ‘multicellular’ protein domains appeared to be more frequently retained in animals than in choanoflagellates (figure 3*a*), showing a very different pattern of conservation of the 5715 protein domains present at the Choanozoan ancestor (figure 3*a*, grey line behind the bar chart). Thus, we interrogated whether these ‘multicellular-animal-like’ protein domains are involved in colony formation in choanoflagellates. Our data show that colonial choanoflagellates have not kept more (16.5 on average) of those protein domains related in multicellular functions than non-colonial taxa (18 on average). This may suggest that the molecular mechanisms involved in animal and choanoflagellate multicellularity require different protein players. One clear example of this is the POU gene, which is present in all animal taxa, but only found in the choanoflagellate *M. fluctuans*.

### (i) UC1 and UC4 SAGs contain protein domains involved in key animal functions

Among the protein domains that originated in Choanozoa, nine protein domains were recovered in our SAGs and also in other choanoflagellate taxa (figure 3*a*). In particular, our UC1 SAG recovered two of the most conserved protein domains in extant animal species: Lamp and TSP_C.

Lamp proteins are uni-domain lysosome-associated membrane glycoproteins, which seem to be required for phagocytic processes and are related to immunogenic responses [92]. Besides UC1, Lamp proteins are more conserved among Craspedida species within choanoflagellates (figure 3). TSP_C is the C terminal domain of thrombospondin proteins, which are secreted proteins that interact with the extracellular matrix and plasma proteins. Thrombospondins are related to embryonic development, tissue differentiation, tumour growth and angiogenesis [93]. Choanoflagellates contain only the C-terminal domain; the full protein architecture is animal-specific.

We also found other protein domains on UC1, which are highly conserved among animal species. Those are Sema and NKAIN (Sema, 20 out of 21 species; NKAIN, 18 out 21 species), both involved in extracellular receptors or transmembrane proteins that participate in neural functions in animals. NKAIN is a sodium-dependent ATPase interacting protein [94], while Sema is the core domain of semaphorins and their binding receptors, Plexin proteins. Semaphorins and Plexins are singalling extracellular proteins involved in the guidance of axon formation in neural development [95]. A recent phylogenetic reconstruction of Semaphorins and Plexins showed the pre-metazoan origin of this domain, which was duplicated before the origin of Choanozoa [96]. Interestingly, the Sema domain found in the SAG UC1 belongs to a Plexin-like protein (electronic supplementary material, figure S7). Therefore, Plexins, semaphorins, and NKAIN proteins predate the origins of animals, together with other genes related in neural functions such as sodium [97] and calcium channels [98], neuroglobulins [99], and proteins related in synapsis [10,100] and neural secretion [101].

Finally, our SAGs contain two protein domains gained at Choanozoa that are less conserved in all metazoan species: NPM1-C and Fanconi_A_N (figure 3). NPM1-C is the C-terminal protein domain of the transcription factor Nucleophosmin, previously thought to be vertebrate-specific [102]. NPM1-C is essential in the regulation of DNA replication malfunctions, and it is involved in p53-mediated pathways to promote apoptosis in case of DNA damage [102]. Nucleophosmin protein domain architecture consists of the Nucleoplasmin protein domain followed by NPM1-C in all animals. The domain Nucleoplasmin is paneukaryotic, and the protein domain architecture consisting of Nucleoplasmin plus NPM1-C is animal-specific according to our results. In the SAG UC4 we identified the domain NPM1-C, which we confirmed by phylogeny not to be a contamination (electronic supplementary material, figure S8), while the Nucleoplasmin domain was missing. As SAGs genomes are partial, we cannot rule out the possibility that the Nucleoplasmin domain is indeed present in this taxa. However, we believe the most likely explanation is that the animal Nucleophosmin, as Smad proteins, appeared as a product of a domain shuffling between the two more ancient Nucleophosmin domains (Nucleoplasmin and NPM1-C) at the origin of animals. It may not be essential in maintaining animal functions, given its low conservation among extant animal species (30%), but it might be crucial to perform vertebrate-specific functions because it is conserved among all analysed vertebrates.

Fanconi_A_N is the N-terminal domain of the Fanconi anaemia complementation group A protein (FANCA human protein) that acts in DNA damage-repair processes and also in the differentiation of blood cells [103]. Mutations in these gene cause Fanconi anaemia (FA) in humans [103]. Our data show a pre-metazoan origin for the Fanconi_A_N domain, and a vertebrate acquisition for the Fanconi_A domain that, together with Fanconi_A_N, conform the canonical FANCA protein. This is probably the reason why this protein domain, like Nucleophosmin, is conserved in all vertebrate species.

Thus, our SAGs from uncultured choanoflagellate species, UC1 and UC4, together with the new choanoflagellate transcriptomic data [23] have allowed us to show the pre-metazoan origin of protein domains, present in overlooked proteins that could have been essential in the origins of animals and, therefore, in the transition towards animal multicellularity. These include Lamp, thrombospondins, NKAIN, Semaphorins and Plexin proteins. Finally, we also identified proteins that are particularly conserved among vertebrate species but not in other metazoans, like Nucleophosmin and FANCA protein.

## 4. Discussion

In this work, we took advantage of the single-cell genomics technique to expand the extant genomic diversity of choanoflagellates by recovering a substantial proportion of the genomes of two uncultured choanoflagellate species (UC1 and UC4). Our data, together with the recent new genomic/transcriptomic information from 19 choanoflagellates and other unicellular holozoans [14,23,41], have allowed us to perform, with an unprecedented level of detail, the reconstruction of the protein domains' gains and losses from the LECA to the Urmetazoa, improving our understanding of the genomic changes that allowed the transition towards multicellularity in animals.

As commented in previous studies, the current state of single-cell genomics techniques based on cell isolation, cell lysis and whole genome amplification with MDA, has important limitations in terms of genome recovery [34,40]. Our results are in agreement with the literature: only two out of our four SAG assemblies (UC1 and UC4) contained enough genomic information (31.7–13.5% BUSCO completeness values) to perform gene and protein domain annotation. These limitations have a strong effect on genome-wide analyses of gene evolution based on SAG data. For instance, gene-level comparative analyses are strongly affected by the incompleteness of SAG assemblies. Similarly, SAG-derived gene predictions cannot be used in probabilistic ancestral reconstructions of gene content that rely on estimations of gain and loss rates at different branches (e.g. [72]) like, for instance, our estimations of gene retention probabilities in various clades (figure 4). In this case, the inclusion of UC1 and UC4 in this analysis would have had underestimated the probability of gene retention in the wider choanoflagellate clade.

Thus, genome-wide comparative analyses using SAG data need to be designed aiming to minimize the effects of incompleteness biases. For example, gene family evolutionary studies can be based on protein domains instead of full-length genes (to take advantage of the fact that the recovery rate of individual protein domains from SAG assemblies is higher than for genes [34]). In addition, our comparative framework complemented our SAGs with a wide array of complete genomic and transcriptomic data from other choanoflagellates (21 in total) [9,10,23] and other eukaryotes. By taking these limitations into account in our experimental design, we were able to take leverage of the limited genomic information contained in UC1 and UC4 in two key analyses: our phylogenomic analysis, which includes the widest sampling of choanoflagellates to date (figure 2), and the reconstruction of ancestral protein domain evolution from the LECA to the origin of animals (figure 3). This ancestral reconstruction, based on Dollo parsimony, led to the identification of a Nucleophosmin-linked domain as evolving in the Choanozoan ancestor, which could be confirmed using phylogenetic analysis (electronic supplementary material, figure S7). Thus, single-cell genomics data, with its limitations, can be a good resource for phylogenomics and also for gene family evolutionary studies.

From the protein domain evolutionary analysis, we show that the high degree of genic innovation that occurred at the stem of Metazoa did not coincide with an important increase in protein domain richness. Instead, while the Urmetazoan ancestor acquired a large number of new gene families, it contained fewer protein domains than its unicellular Choanozoan ancestor. There are two reasons for this. First, because animals lost many genes related to metabolic functions mainly involved in amino acid biosynthesis and carbohydrate metabolism, implying an important change in their metabolic and ecological niche capabilities [23]. Second, many new gene families were the result of the combined action of gene duplications and domain shuffling events [14], which originated the important increase of around 1500–1700 new gene families at stem of Metazoa [22,23].

We have also described many examples of protein domains that appeared before the transition to animal multicellularity and that are highly conserved among animals. However, when encoded in pre-metazoan proteins, they do not present the animal-like protein domain architectures. This is the case, for example, for Nucleophosmin or the nuclear hormone receptor. Thus, domain shuffling events that were already described to explain the appearance of Notch [9,12], or more recently, Smad proteins [23], seem to be the mechanism by which the unicellular ancestor of animals rearranged its genome as it progressed towards animal multicellularity, together with domain duplications. However, we cannot discard the less parsimonious possibility that the Choanozoan ancestor had a much more complete set of protein architectures than animals that has been lost in the lineage leading to extant choanoflagellates. Interestingly, the choanoflagellate *M. fluctuans* has a clear Notch homologue with the prototypical EGF, Notch transmembrane and Ank domains in canonical order [23].

Regarding our analysis of the evolutionary retention of protein domains, we show that the gains acquired in the unicellular holozoan ancestors (Holozoa, Filozoa, Choanozoa) were, proportionally, more retained by animals than by their unicellular counterparts (figure 4). However, this can be biased by the poor knowledge that we have of the unicellular relatives of animals, given that specific protein domains acquired in the unicellular holozoan ancestors, which were subsequently lost or poorly conserved in animals, would be very unlikely to appear in the Pfam database. As it has been shown that choanoflagellates contain a rich set of specific genes [23], this situation might be affecting our results.

Following this rationale, the coloniality in choanoflagellates would be largely based on genetic innovations without direct homology to animal genes, as both multicellular stages (choanoflagellate colonies and clonal multicellularity of animals) might had been facilitated by different genetic players. This comparative genomic approach is in agreement with previous transcriptomic analyses that showed that the colonial stage of *S. rosetta* is enriched in evolutionarily recent choanoflagellate-specific genes [10]. Thus a deeper, comprehension of choanoflagellate gene functions is required to understand the subtle homology relationships between animal multicellularity and choanoflagellate coloniality.

Finally, our analysis has shown that POU genes were already present before the transition towards animal multicellularity. POU is a transcription factor involved in development and the maintenance of undifferentiated cells [89]. Thus, it is unknown which functions were involved in the Choanozoan ancestor. Together with POU, we revealed other overlooked proteins whose canonical protein domain architecture is highly conserved among metazoans, like Lamp or thrombospondin proteins. One could argue that Lamp proteins (lysosomal membrane proteins that have been related with phagocytosis [92]) could have been key in improving the predatory capabilities of the unicellular metazoan ancestor. These new predatory capabilities could have been crucial in the establishment of animal multicellularity, especially whether it is taken into account that bacterial cues can induce life cycle transitions in choanoflagellates [104,105].

## 5. Conclusion

Overall, in this work we have revealed the genomic changes that facilitated the origins of animal multicellularity. These include massive domain shuffling events and duplications events of pre-existent protein components, discrete acquisition of new domains and the loss of metabolic proteins. We have also described the composition of the Urmetazoan and several unicellular ancestors of animals, showing—and in some cases redefining—the evolutionary origin of the most essential protein domains required for animal multicellularity. Finally, we revealed that proteins extremely conserved in all animal species are not limited to the usual suspects involved in functions such as signalling, adhesion or gene expression regulation (like wnt, integrins or Ets transcription factors). Indeed, there is a wider spectrum of overlooked protein domains involved in neural functions, nuclear organization, phagocytosis or without a known-associated function, which are highly conserved among extant animals species. Thus, we here have expanded the knowledge of new putative genetic players required for the emergence of animal multicellularity.

## Supplementary Material

Supplementary Tables and Figures

## Data accessibility

Raw reads can be found at ENA, project accession number PRJEB31614. SAGs assemblies and annotations, protein domain matrix, summary of protein domain gains in different Opisthokonta ancestors, metazoan losses information and all the supplementary information of the phylogenetic analysis can be found at Figshare (https://doi.org/10.6084/m9.figshare.7819571.v1).
