# Supplementary material for: Reconstruction of protein domain evolution using single-cell amplified genomes of uncultured choanoflagellates sheds light on the origin of animals
Source: Philos Trans R Soc Lond B Biol Sci. 2019 Oct 7;374(1786):20190088. doi: 10.1098/rstb.2019.0088 (PMC6792448; doi:10.1098/rstb.2019.0088)
Supplement: Supplementary Tables and Figures [file rstb20190088supp1.pdf]

Supplementary material of the following research article submitted to Philosophical transactions of the Royal Society B

Reconstruction of protein domain evolution using single-cell amplified genomes of uncultured choanoflagellates sheds light into animal origins

David López-Escardó[1*], Xavier Grau-Bové[1,2] Amy Guillaumet-Adkins[3,4], Marta Gut[3,4], Michael E. Sieracki[5] & Iñaki Ruiz-Trillo[1,2,6*]

[1]*Institut de Biologia Evolutiva (CSIC-Universitat Pompeu Fabra), Passeig Marítim de la Barceloneta 37-49, 08003 Barcelona, Catalonia, Spain.*

[2]*Departament de Genètica, Microbiologia i Estadística, Universitat de Barcelona, Barcelona, Catalonia, Spain.*

[3]*CNAG-CRG, Centre for Genomic Regulation (CRG), Barcelona Institute of Science and Technology (BIST), Barcelona, Spain.*

[4]*Universitat Pompeu Fabra (UPF), Barcelona, Spain.*

[5]*National Science Foundation; Arlington, VA, USA*

[6] *ICREA, Pg. Lluís Companys 23, 08010 Barcelona.*

*corresponding author: David López-Escardó and Iñaki Ruiz-Trillo

Supplementary Table 1. Main environmental features of the SAG samples:

SAG	SAG TARA ID	TARA station	Coordinates	Date	Depth (m)	Temp (ºC)	Oxygen (µmol/kg)	Salinity (psu)	Chloroph-yll (mg Chl/m3)
UC1	AAA538_N18_CHOA	23	42.1735ºN 17.7252ºE	18-11-09	55	16.9	226.2	38.3	0.19
UC2	AB242_J22_CHOA	51	21.5043º S 42.1735ºE	11-5-10	5.0	27.3	194.3	35.1	0.22
UC3	AB537_J22	41	14.5536º N 70.0128ºE	30-3-10	60	29.1	185.5	36.2	0.45
UC4	AB240_J14	41	14.6059º N 69.9776ºE	30-3-10	5	29.1	187.7	36.2	0.32

Supplementary Table 2. Read information and genome statistics from the assemblies performed in each SAG

SAG	Number of reads	Assembly length (Mb)	GC (%)	N50	L75	Largest scaffold (bp)	Number of scaffolds*
**UC1**	1.24E+08	7.75	49.8	4,928	1,061	41,637	3,276
**UC2**	4.48E+07	1.00	30.8	1,499	351	32,189	746
**UC3**	4.25+07	1.32	33.5	2,197	309	111,870	819
**UC4**	1.15E+08	7.25	40.0	11,360	496	72,672	2,527

Supplementary Table 3. Sumary of the genes annotated in the UC2 mitochondrial genome

**UC2 mitochondrial genes**	
atp9	rps12
cob	rps13
cox1	rps3
cox2	rps4
cox3	rps8
nad1	rrn5
nad2	trnA(ugc)
nad3	trnC(gca)
nad4	trnD(guc)
nad4L	trnE(uuc)
nad5	trnF(gaa)
nad6	trnG(ucc)
orf109	trnH(gug)
orf130	trnI(gau)
orf139	trnK(uuu)
orf143	trnL(uaa)
orf154	trnL(uag)
orf181	trnM(cau)
orf194	trnN(guu)
orf212	trnP(ugg)
orf386	trnQ(uug)
orf408	trnR(ucg)
orf717	trnR(ucu)
rnl	trnS(gcu)
rns	trnS(uga)
rpl14	trnT(ugu)
rpl16	trnV(uac)
rpl2	trnW(uca)
rpl5	trnY(gua)
rpl6	

Supplementary Table 4. Blast identity of SAGs 18S ribosomal sequences againts TARA oceans OTUs

SAG	OTU_code	Id (%)	Nº of missmatches
**UC1**	9b38ebc15ad3400e51b8fdcb3be290e6	100	0
**UC2**	ee0099358d26055b2c572ea2605cdc05	100	0
**UC3**	8da960f4921d51c0e3c8b2fdea2f6330	99.2	1
**UC4**	1e98f1edca3c7471c2f8fa0b3a12cee8	99.2	1

Supplementary Table 5. Sumary of the sequences used for 18S ribosomal gene phylogeny

Nickname	Taxa	SSU
	**Choanoflagellatea**	
CHO_Aung	*Acanthocorbis unguiculata* (Thomsen) Hara et Takahashi	HQ026764
CHO_Aspe	*Acanthoeca spectabilis* Ellis (ATCC PRA-103)	KT757415
CHO_Cper	*Choanoeca perplexa* Ellis (ATCC 50453)	KT757437
CHO_Cbot	*Codosiga botrytis* (Ehrenberg 1838) Stein 1878	JF706243
CHO_Cnat	*Calliacantha natans*	KU587842
CHO_Clon	*Calliacantha longicaudata*	KU587840
CHO_Chol	*Codosiga hollandica Carr, Richter and Nitsche (ATCC PRA-388)*	KT757430
CHO_Csp1	*Codosiga sp. M1/pIIp*	JF706237
CHO_Csp2	*Codosiga sp. M2/Morocco*	JF706236
CHO_Csp3	*Codosiga sp. M3/Mvid*	JF706242
CHO_Csp5	*Codosiga sp. M5/Iceland*	JF706239
CHO_Desp	*Desmarella sp.*	AF084231
CHO_Dgra	*Diaphanoeca grandis Ellis (ATCC 50111)*	KT757448
CHO_Dsph	*Diaphanoeca sphaerica*	KU587846
CHO_Disp	*Diaphanoeca sp.*	HQ237460
CHO_Dcos	*Didymoeca costata (Valkanov) Doweld (ATCC PRA-389)*	KT757444
CHO_Hbal	*Hartaetosiga balthica (Wylezich et Karpov) Carr, Richter and Nitsche (ATCC 50964)*	KT757421
CHO_Hgra	*Hartaetosiga gracilis (Kent) Carr, Richter and Nitsche (ATCC 50454)*	KT757426
CHO_Hmin	*Hartaetosiga minima (Wylezich et Karpov) Carr, Richter and Nitsche*	JQ034422
CHO_Hnan	*Helgoeca nana Leadbeater (ATCC 50073)*	KT757452
CHO_Mroa	*Microstomoeca roanoka (ATCC 50931) Carr, Richter and Nitsche*	KT757502
CHO_Mbre	*Monosiga brevicollis Ruinen (ATCC 50154)*	AF084618
CHO_Mflu	*Mylnosiga fluctuans Carr, Richter and Nitsche (ATCC 50635)*	AF084230
CHO_Pmin	*Pleurasiga minima*	KU587849
CHO_Pped	*Parvicobicula pedunculata* Leadbeater	HQ026765
CHO_Prey	*Pleurasiga reynoldsii*	KU587851
CHO_Pdic	*Polyoeca dichotoma* Kent (*Calliacantha* sp. CEE-2003)	AF272000
CHO_Scal	*Salpingoeca calixa* Carr, Richter and Nitsche	KT757470
CHO_Sdol	*Salpingoeca dolichothecata* (ATCC 50959) Carr, Richter and Nitsche	KT757472
CHO_Seur	*Salpingoeca euryoecia* Jeuck, Arndt & Nitsche	KJ631038

CHO_Sfus	*Salpingoeca fusiformis* Kent	KJ631044
CHO_Shel	*Salpingoeca helianthica* (ATCC 50153) Carr, Richter and Nitsche	KT757487
CHO_Sinf	*Salpingoeca infusionum* Kent (ATCC 50559)	KT757477
CHO_Slon	*Salpingoeca longipes* Kent	KJ631046
CHO_Smac	*Salpingoeca macrocollata* (ATCC 50938) Carr, Richter and Nitsche	KT757482
CHO_Soah	*Salpingoeca oahu* Carr, Richter and Nitsche	KT757492
CHO_Spun	*Salpingoeca punica* (ATCC 50788) Carr, Richter and Nitsche	KT757460
CHO_Skve	*Salpingoeca kvevrii* (ATCC 50929) Carr, Richter and Nitsche	KT757494
CHO_Sros	*Salpingoeca rosetta* King (ATCC 50818)	EU011924
CHO_Stub	*Salpingoeca tuba* Kent	HQ026774
CHO_Surc	*Salpingoeca urceolata* Kent (ATCC 50560)	KT757514
CHO_Sven	*Salpingoeca ventriosa* Jeuck, Arndt and Nitsche	KJ631041
CHO_Spar	*Savillea parva* Norris (ATCC PRA-391)	KT757467
CHO_Slep	*Sphaeroeca leprechaunica* Jeuck, Arndt & Nitsche	KJ631047
CHO_Svol	*Sphaeroeca volvox* Lauterborn	Z34900
CHO_Spyr	*Stagondoeca pyriformis* Carr, Richter and Nitsche	KT757499
CHO_Sarn	*Stephanoeca arndtii* Nitsche	JX069943
CHO_Saph	*Stephanoeca apheles* Thomsen	EF523336
CHO_Scau	*Stephanoeca cauliculata* Leadbeater	HQ026766
CHO_Sdip	*Stephanoeca diplocostata* Ellis (ATCC PRA-392)	KT757508
CHO_Snor	*Stephanoeca norrisii* Thomsen	HQ026768
CHO_Spau	*Stephanoeca paucicostata* Throndsen	HQ026769
DQ995807_Lagenoeca_artica	*Lagenoeca artica* Nitsche 2007	DQ995807
	**Uncultured Holozoans**	
HQ219444_FRESCHO1	FRESCHO1 del Campo 2013	HQ219444
AY821948_FRESCHO4	FRESCHO4 del Campo 2013	AY821948
AY821949_Ukn	FRESCHO3 del Campo 2013	AY821949
GU647170_CladeL	Clade L Weber 2012	GU647170
EF024885_CladeL	Clade L Weber 2012	EF024885
GU825407_ChoanoflagellateE	MACHO1 del Campo 2013	GU825407
JQ223245_Ukn	Unassinged Acanthoecida del Campo 2013	JQ223245
DQ104587_FRESCHO1	FRESCHO1 del Campo 2013	DQ104587
FJ410610_FRESCHO2	FRESCHO2 del Campo 2013	FJ410610
GU647190_FRESCHO2	FRESCHO2 del Campo 2013	GU647190
AY426842_Lagenoeca_ENV	Lagenoeca del Campo 2013	AY426842
FJ176220_1_MAOP_1	MAOP1 del Campo 2013	FJ176220
GU825148_MAOP1	MAOP1 del Campo 2013	GU825148
GU385597_1_MAOP_2	MAOP2 del Campo 2013	GU385597

GU824782_1_MAOP2	MAOP2 del Campo 2013	GU824782
AB191435_1_MAIP1_Ukn	MAIP1 del Campo 2013	AB191435
HQ219425_1_FRESHIP1_Ukn	FRESHIP1 del Campo 2013	HQ219425
DQ244007_1_FRESHIP2_Ukn	FRESHIP2 del Campo 2013	DQ244007
UC1	UC1 Clade 1 Craspedida Lopez-Escardo 2017	MN028775
UC2	UC2 Acanthoecidae Lopez-Escardo 2017	MN028774
UC3	UC3 Stephanocidae Lopez-Escardo 2017	MN028780
UC4	UC4_Early branching Acanthoecida Lopez-Escardo 2017	MN028777
	**Filasterea**	
Opistho_2_18S	*Pigoraptor chileana* Hehenberger 2017	MF190553
LAB0002_Opistho1	*Pigoraptor vitenamica* Hehenberger 2017	MF190552
AF436888_1_Capsaspora_owczarzaki	*Capsaspora owczarzaki*	AF436888
AF271998_1_Ministeria_vibrans	*Ministeria vibrans*	AF271998
	**Ichthyosporea**	
Apar	*Amoebidium parasiticum* Cienkowski	Y19155
Ihof	*Ichthyophonus hoferi* Plehn & Mulsow	U25637
LAB0004_NK52	*Chromosphaera perkinsii* Grau-Bové 2017	MN065157
FN996945_1_Sphaerothecum_destruens	*Sphaerothecum destruens*	FN996945
AY363958_1_Anurofeca_sp_LAH_2003	*Anurofeca sp.*	AY363958
AY336701_1_Eccrinales	*Eccrinales*	AY336701
EU124916_1_Creolimax_fragrantissima	*Creolimax fragrantissima*	EU124916
GU810144_1_Pirum_gemmata	*Pirum gemmata*	GU810144
GU810145_1_Abeoforma_whisleri	*Abeoforma whisleri*	GU810145
Y16260_2_Sphaeroforma_arctica	*Sphaeroforma arctica*	Y16260_2
AF533941_1_Dermocystidium_percae	*Dermocystidium percae*	AF533941
AY372365_1_Rhinosporidium_sp	*Rhinosporidium sp.*	AY372365
	**Plurimorfea**	
L42528_1_Corallochytrium_limacisporum	*Corallochytrium limacisporum*	L42528
LAB0003_Colp12	*Syssomonas multiformis* Hehenberger 2017	MF190551
	**Metazoa**	
Bova	*Beroe ovata* Mayer	AF293694
Hasp	*Halichondria* sp.	AY737639
Hlsp	*Haliclona* sp.	AY734450
Lesp	*Leucosolenia* sp.	AF100945
Nvec	*Nematostella vectensis* Stephenson	AF254382
Susp	*Suberites* sp.	AF100947
Sysp	*Sycon* sp.	AM180970
Tadh	*Trichoplax adhaerens* von Schultze	AY652581
AF102892_Acoela_Paratomella_rubra	*Paratomella rubra*	AF102892
AY040680_Acanthobdella_peledina	*Acanthobdella peledina*	AY040680
U49909_Milnesium_tardigradum	*Milnesium tardigradum*	U49909
AJ228794_Pseudoceros_tritriatus	*Pseudoceros tritriatus*	AJ228794
EU368616_Ctenolepisma_longicaudata	*Ctenolepisma longicaudata*	EU368616
D14357_Antedon_serrata	*Antedon serrata*	D14357

D14359_Balanoglossus_carnosus	*Balanoglossus carnosus*	D14359
AF120533_Mollusca_Lima_lima	*Lima lima*	AF120533
AY049861_Urobatis_jamaicensis	*Urobatis jamaicensis*	AY049861
	**Holomycota**	
LAB0001_Parvularia_sp_ATCC50694	*Parvularia atlantis* López-Escardó 2017	KY113120
AB433328_Nuclearia_thermophila	*Nuclearia thermophila* Yoshida 2009	AB433328
FJ816018_Fonticula_alba	*Fonticula alba*	FJ816018
AY546684_Spizellomyces_punctatus	*Spizellomyces punctatus*	AY546684
DQ536481_1_Cyllamyces_aberensis	*Cyllamyces aberensis*	DQ536481
KC673103_1_Malassezia_globosa	*Malassezia globosa*	KC673103

Supplementary Table 6. Sumary of the taxa and the genes used in the eight-gene phylogeny. All the sequences are available at Figshare.

Nickname	Taxa	SSU	LSU	hsp90	tubA	EFL	EF-1A	Actin	hsp70
	**Choanoflagellatea**								
Aung	*Acanthocorbis unguiculata* (Thomsen) Hara et Takahashi	HQ026764	-	-	-	-	-	-	-
Aspe	*Acanthoeca spectabilis* Ellis (ATCC PRA-103)	KT757415	KT757416	KT757419	KT757420	KT757418	KT757417	comp16397_c1_seq5_1_7.8e257	comp16977_c0_seq1_1_2.7e292
Cper	*Choanoeca perplexa* Ellis (ATCC 50453)	KT757437	KT757438	KT757435	KT757439	KT757434	-	comp25775_c0_seq1_1_3.5e195	comp12883_c0_seq1_1_0
Cbot	*Codosiga botrytis* (Ehrenberg 1838) Stein 1878	JF706243	KT757422	-	-	-	HQ896019	HQ896017.1	HQ896020
Chol	*Codosiga hollandica* Carr, Richter and Nitsche (ATCC PRA-388)	KT757430	KT757431	KT757433	KT757436	-	KT757432	comp52078_c1_seq1_1_9.6e172	comp56372_c0_seq1_1_0
Csp1	*Codosiga* sp. M1/pIIp	JF706237	KT757440	-	-	-	-	-	-
Csp2	*Codosiga* sp. M2/Morocco	JF706236	KT757441	-	-	-	-	-	-
Csp3	*Codosiga* sp. M3/Mvid	JF706242	KT757442	-	-	-	-	-	-
Csp5	*Codosiga* sp. M5/Iceland	JF706239	KT757443	-	-	-	-	-	-
Desp	*Desmarella* sp.	AF084231	-	-	-	-	-	-	-
Dgra	*Diaphanoeca grandis* Ellis (ATCC 50111)	KT757448	EU011939	KT757450	KT757451	KT757449	KT768098	comp33416_c0_seq1_1_4.2e238	comp25564_c0_seq1_1_5.7e268
Disp	*Diaphanoeca* sp.	HQ237460	-	-	-	-	-	-	-
Dcos	*Didymoeca costata* (Valkanov) Doweld (ATCC PRA-389)	KT757444	EU011938	KT757446	KT757447	KT757445	-	comp16066_c1_seq2_1_1.5e138	comp16248_c0_seq2_1_3.1e245 1920
Hbal	*Hartaetosiga balthica* (Wylezich et Karpov) Carr, Richter and Nitsche (ATCC 50964)	KT757421	KT988065	KT757424	KT757425	KT757423	-	comp2182_c0_seq1_1_6.5e253	comp9438_c2_seq1_1_4.7e296

Hgra	*Hartaetosiga gracilis* (Kent) Carr, Richter and Nitsche (ATCC 50454)	KT757426	EU011935	KT757428	KT757429	KT757427	-	comp11731_c0_seq1_1_1.3e257	comp13062_c2_seq4_substitute
Hmin	*Hartaetosiga minima* (Wylezich et Karpov) Carr, Richter and Nitsche	JQ034422	JQ034423	-	-	-	-	-	-
Hnan	*Helgoeca nana* Leadbeater (ATCC 50073)	KT757452	KT757453	KT768096	KT768097	KT757455	KT757454	comp25775_c0_seq1_1_3.5e195	comp27130_c3_seq9_1_9.3e285
Mroa	*Microstomoeca roanoka* (ATCC 50931) Carr, Richter and Nitsche	KT757502	KT757503	KT757505	KT757506	KT757504	-	comp15624_c0_seq2_1_2.5e257	comp16714_c3_seq1_1_0
Mbre	*Monosiga brevicollis* Ruinen (ATCC 50154)	AF084618	KT757456	AY226081	AY026070	AY026073	-	37852_1_5.2e269	35238_1_0
Mflu	*Mylnosiga fluctuans* Carr, Richter and Nitsche (ATCC 50635)	AF084230	EU011940	KT757458	KT757459	-	KT757457	comp10041_c0_seq1_1_4.9e219	comp10254_c0_seq1_1_0
Pped	*Parvicobicula pedunculata* Leadbeater	HQ026765	-	-	-	-	-	-	-
Pdic	*Polyoeca dichotoma* Kent (*Calliacantha* sp. CEE-2003)	AF272000	-	-	-	-	-	-	-
Scal	*Salpingoeca calixa* Carr, Richter and Nitsche	KT757470	KT757471	-	-	-	-	-	-
Sdol	*Salpingoeca dolichothecata* (ATCC 50959) Carr, Richter and Nitsche	KT757472	KT757473	KT757475	KT757476	-	KT757474	comp26491_c0_seq1_1_2.9e179	comp26705_c0_seq1_1_2.8e275
Seur	*Salpingoeca euryoecia* Jeuck, Arndt & Nitsche	KJ631038	-	-	-	-	-	-	-
Sfus	*Salpingoeca fusiformis* Kent	KJ631044	-	-	-	-	-	-	-
Shel	*Salpingoeca helianthica* (ATCC 50153) Carr, Richter and Nitsche	KT757487	KT757488	KT757490	KT757491	-	KT757489	comp15346_c0_seq1_1_1.9e266	comp15440_c5_seq1_1_5e192
Sinf	*Salpingoeca infusionum* Kent (ATCC 50559)	KT757477	KT757478	KT757480	KT757481	KT757479	-	comp14544_c0_seq3_1_3.8e262	comp11421_c0_seq1_1_0

Slon	*Salpingoeca longipes* Kent	KJ631046	-	-	-	-	-	-	-
Smac	*Salpingoeca macrocollata* (ATCC 50938) Carr, Richter and Nitsche	KT757482	KT757483	KT757485	KT757486	KT757484	-	comp22230_c1_seq2_1_1.6e254	comp14111_c0_seq1_1_0
Soah	*Salpingoeca oahu* Carr, Richter and Nitsche	KT757492	KT757493	-	-	-	-	-	-
Spun	*Salpingoeca punica* (ATCC 50788) Carr, Richter and Nitsche	KT757460	KT757461	KT757464	KT757469	KT757463	KT757462	comp16274_c1_seq2_1_9.8e253	comp16816_c2_seq2_1_1.5e238
Skve	*Salpingoeca kvevrii* (ATCC 50929) Carr, Richter and Nitsche	KT757494	EU011926	KT757497	KT757498	KT757496	-	comp10800_c0_seq1_1_3.3e247	comp11028_c1_seq14_1_0
Sros	*Salpingoeca rosetta* King (ATCC 50818)	EU011924	EU011941	KT757501	AY226048	XM_004996684	-	PTSG_01553T0_1_3.1e259	PTSG_10966T0_1_0
Stub	*Salpingoeca tuba* Kent	HQ026774	KT757507	-	-	-	-	-	-
Surc	*Salpingoeca urceolata* Kent (ATCC 50560)	KT757514	KT757515	KT757517	KT757516	KT757518	-	comp19597_c0_seq4_1_1.3e249	comp17673_c0_seq6_1_1.3e298
Sven	*Salpingoeca ventriosa* Jeuck, Arndt and Nitsche	KJ631041	KT757519	-	-	-	-	-	-
Spar	*Savillea parva* Norris (ATCC PRA-391)	KT757467	KT757495	KT757465	KT757466	KT757468	-	comp11563_c0_seq1_1_7.6e253	comp11748_c0_seq1_1_5.2e297
Slep	*Sphaeroeca leprechaunica* Jeuck, Arndt & Nitsche	KJ631047	-	-	-	-	-	-	-
Svol	*Sphaeroeca volvox* Lauterborn	Z34900	-	-	-	-	-	-	-
Spyr	*Stagondoeca pyriformis* Carr, Richter and Nitsche	KT757499	KT757500	-	-	-	-	-	-
Sarn	*Stephanoeca arndtii* Nitsche	JX069943	-	-	-	-	-	-	-
Saph	*Stephanoeca apheles* Thomsen	EF523336	-	-	-	-	-	-	-
Scau	*Stephanoeca cauliculata* Leadbeater	HQ026766	-	-	-	-	-	-	-

Sdip	*Stephanoeca diplocostata* Ellis (ATCC PRA-392)	KT757508	KT757509	KT757512	KT757513	KT757511	KT757510	comp19908_c2_seq1_1_8.2e131	comp18193_c0_seq1_1_1.4e275
Snor	*Stephanoeca norrisii* Thomsen	HQ026768	-	-	-	-	-	-	-
Spau	*Stephanoeca paucicostata* Throndsen	HQ026769	-	-	-	-	-	-	-
	**Undescribed Choanoflagellatea**								
UC1		MN028775	XXXXX	-	-	-	XXXXX	XXXXX	XXXXX
UC2		MN028774	XXXXX	-	-	-	-	XXXXX	-
UC3		MN028780	XXXXX	-	-	-	-	-	-
UC4		MN028777	XXXXX	-	-	XXXXX	-	XXXXX	XXXXX
	**Ichthyosporea**								
Apar	*Amoebidium parasiticum* Cienkowski	Y19155	EU011932	Apar_MIRA_nonfilt_contigs.fasta Apar_ReadsTotals_rep_c25415	-	-	AY582828	-	-
Ihof	*Ichthyophonus hoferi* Plehn & Mulsow	U25637	AY026370	-	-	-	AF450116 (*I. irregularis*)	-	-
	**Metazoa**								
Bova	*Beroe ovata* Mayer	AF293694	AY026369	-	-	-	-	-	-
Hasp	*Halichondria* sp.	AY737639	-	AY226083	AY226049	-	GQ330929	-	-
Hlsp	*Haliclona* sp.	AY734450	AF441340	AY226084	AY226050	-	JQ606691	-	-
Lesp	*Leucosolenia* sp.	AF100945	AY026372	AY226087	AY226053	-	DQ087468	-	-

Nvec	*Nematostella vectensis* Stephenson	AF254382	Genome GCA_000209225.1 contig NEMVEscaffold_301 dna:supercontig:GCA000209225.1: NEMVEscaffold_310:1:244201:1	AY226090	AY226056	-	XM_001625261	-	195315_1_3.4e258
Susp	*Suberites* sp.	AF100947	AY026381	AY226085	AY226051	-	GQ330984	-	-
Sysp	*Sycon* sp.	AM180970	AM181000	AY226088	AY226054	-	DQ087461	-	-
Tadh	*Trichoplax adhaerens* von Schultze	AY652581	AY303975	XM_002113141	XM_002110626	-	NW_002060962	63375_1_6.9e197	38294_1_1.5e216

Supplementary Table 7. Summary of eukaryotic species used for the comparative genomics analysis

Species	Taxonomy	Abbreviation	Reference
Homo sapiens	Metazoa	Hsap	Ensmbl 80
Mus musculus	Metazoa	Mmus	Ensmbl 80
Xenopus tropicalis	Metazoa	Xtro	(Hellsten *et al.* 2010)
Branchiostoma floridae	Metazoa	Bflo	(Putnam *et al.* 2008)
Ciona intestinalis	Metazoa	Cint	Ensmbl 80
Oikopleura dioica	Metazoa	Odio	http://www.genoscope.cns.fr/externe/GenomeBrowser/Oikopleura/
Daphnia pulex	Metazoa	Dpul	Ensmbl Metazoa 27
Drosophila melanogaster	Metazoa	Dmel	Ensmbl Metazoa 27
Tribolium castaneum	Metazoa	Tcas	Ensmbl Metazoa 27
Capitella teleta	Metazoa	Ctel	Ensmbl Metazoa 27
Saccoglossus kowalevskii	Metazoa	Skow	Ensmbl Metazoa 27
Lottia gigantea	Metazoa	Lgig	Ensmbl Metazoa 27
Trichoplax adhaerens	Metazoa	Tadh	Ensmbl Metazoa 27
Nematostella vectensis	Metazoa	Nvec	Ensmbl Metazoa 27
Aiptasia	Metazoa	Aipt	Ensmbl Metazoa 27
Hydra magnipapillata	Metazoa	Hmag	https://research.nhgri.nih.gov/hydra/
Mnemiopsis leidyi	Metazoa	Mlei	Ensmbl Metazoa 27
Acropora digitifera	Metazoa	Adig	https://marinegenomics.oist.jp/coral/viewer/download?project_id=3
Amphimedon queenslandica	Metazoa	Aque	Ensmbl Metazoa 27
Oscarella carmela	Metazoa	Ocar	https://gold.jgi.doe.gov/project?id=76209
Sycon ciliatum	Metazoa	Scil	https://datadryad.org/resource/doi:10.5061/dryad.tn0f3/1
Codosiga hollandica	Choanoflagellata	Chol	(Richter *et al.* 2018)
UC4	Choanoflagellata	UC4	This work
Helgoeca nana	Choanoflagellata	Hnan	(Richter *et al.* 2018)
Didymoeca costata	Choanoflagellata	Dcos	(Richter *et al.* 2018)
Savillea parva	Choanoflagellata	Sepa	(Richter *et al.* 2018)
Acanthoeca spectabilis	Choanoflagellata	Aspe	(Richter *et al.* 2018)
Stephanoeca diplocostata	Choanoflagellata	Sdip	(Richter *et al.* 2018)

Diaphanoeca grandis	Choanoflagellata	Dgra	(Richter *et al.* 2018)
Salpingoeca dolichothecata	Choanoflagellata	Sdol	(Richter *et al.* 2018)
Salpingoeca rosetta	Choanoflagellata	Sros	(Richter *et al.* 2018)
Salpingoeca roanoka	Choanoflagellata	Sroa	(Richter *et al.* 2018)
Hartaetosiga balthica	Choanoflagellata	Hbal	(Richter *et al.* 2018)
Hartaetosiga gracilis	Choanoflagellata	Hgra	(Richter *et al.* 2018)
Salpingoeca infusionum	Choanoflagellata	Sinf	(Richter *et al.* 2018)
Monosiga brevicollis	Choanoflagellata	Mbre	
Choanoeca perplexa	Choanoflagellata	Cper	(Richter *et al.* 2018)
UC1	Choanoflagellata	UC1	This work
Salpingoeca kvevrii	Choanoflagellata	Skve	(Richter *et al.* 2018)
Salpingoeca urceolata	Choanoflagellata	Surc	(Richter *et al.* 2018)
Salpingoeca macrocollata	Choanoflagellata	Smac	(Richter *et al.* 2018)
Salpingoeca punica	Choanoflagellata	Sapu	(Richter *et al.* 2018)
Salpingoeca helianthica	Choanoflagellata	Shel	(Richter *et al.* 2018)
Mylnosiga fluctuans	Choanoflagellata	Mflu	(Richter *et al.* 2018)
Pigoraptor vietnamica	Filasterea	Pvie	(Hehenberger *et al.* 2017)
Pigoraptor chilena	Filasterea	Pchi	(Hehenberger *et al.* 2017)
Capsaspora owczarzaki	Filasterea	Cowc	(Suga *et al.* 2013)
Ministeria vibrans	Filasterea	Mvib	(Torruella *et al.* 2015)
Abeoforma whisleri	Teretosporea	Awhi	(Torruella *et al.* 2015)
Creolimax fragrantissima	Teretosporea	Cfra	(Torruella *et al.* 2015)
Pirum gemmata	Teretosporea	Pgem	(Torruella *et al.* 2015)
Sphaeroforma arctica	Teretosporea	Sarc	(Torruella *et al.* 2015)
Sphaerothecum destruens	Teretosporea	Sdes	(Torruella *et al.* 2015)
Chromosphaera perkinsii	Teretosporea	Nk52	(Grau-Bové *et al.* 2017)
Syssomonas multiformis	Teretosporea	Smul	(Hehenberger *et al.* 2017)
Corallochytrium limacisporum	Teretosporea	Clim	(Grau-Bové *et al.* 2017)
Fonticula alba	Discicristoidea	Falb	https://www.ncbi.nlm.nih.gov/bioproject/189483
Parvularia atlantis.	Discicristoidea	Nspp	(Torruella *et al.* 2015)
Spizellomyces	Chytridiomycota	Spun	(Torruella *et al.* 2015)

punctatus			
Batrachochytrium dendrobatidis	Chytridiomycota	Bden	Ensmbl Fungi 27
Mortierella verticillata	incertae sedis	Mver	Ensmbl Fungi 27
Rozella allomycis	Cryptomycota	Rall	Ensmbl Fungi 27
Encephalitozoon cuniculi	Microsporidia	Ecun	Ensmbl Fungi 27
Nematocida parisii	Microsporidia	Npar	Ensmbl Fungi 27
Piromyces sp. E2	Neocallimastigomycota	Pisp	Ensmbl Fungi 27
Catenaria anguillulae	Blastocladiomycota	Cang	Ensmbl Fungi 27
Allomyces macrogynus	Blastocladiomycota	Amac	Ensmbl Fungi 27
Rhizophagus irregularis	Glomeromycota	Rirr	Ensmbl Fungi 27
Coemansia reversa	Kickxellomycotina	Crev	Ensmbl Fungi 27
Conidiobolus coronatus	Entomophthoromycota	Ccor	Ensmbl Fungi 27
Rhizopus oryzae	Mucoromycotina	Rory	Ensmbl Fungi 27
Gonapodya prolifera	Monoblepharidomyocta	Gpro	Ensmbl Fungi 27
Coprinopsis cinerea	Basidiomycota	Ccin	Ensmbl Fungi 27
Cryptococcus neoformans	Basidiomycota	Cneo	Ensmbl Fungi 27
Ustilago maydis	Basidiomycota	Umay	Ensmbl Fungi 27
Neurospora crassa	Ascomycota	Ncra	Ensmbl Fungi 27
Saccharomyces cerevisiae	Ascomycota	Scer	Ensmbl Fungi 27
Schizosaccharomyces pombe	Ascomycota	Spom	Ensmbl Fungi 27
Thecamonas trahens	Apusozoa	Ttra	(Torruella *et al.* 2015)
Pygsuia biforma	Breviatea	Pbif	(Torruella *et al.* 2015)
Acanthamoeba castellanii	Amoebozoa	Acas	Ensmbl Protist 27
Dictyostelium discoideum	Amoebozoa	Ddis	Ensmbl Protist 27
Entamoeba histolytica	Amoebozoa	Ehis	Ensmbl Protist 27
Polysphondylium pallidum	Amoebozoa	Ppal	Ensmbl Protist 27
Physarum polycephalum	Amoebozoa	Ppol	Ensmbl Protist 27
Perkinsus marinus	Alveolata	Pmar	Ensmbl Protist 27
Paramecium tetraurelia	Alveolata	Ptet	Ensmbl Protist 27
Symbiodinium minutum	Alveolata	Smin	Ensmbl Protist 27
Toxoplasma gondii	Alveolata	Tgon	Ensmbl Protist 27
Tetrahymena	Alveolata	Tthe	Ensmbl Protist 27

thermophila			
Aplanochytrium kerguelense	Heterokonta	Aker	Ensmbl Protist 27
Aurantiochytrium limacinum	Heterokonta	Alim	Ensmbl Protist 27
Ectocarpus siliculosus	Heterokonta	Esil	Ensmbl Protist 27
Phytophthora infestans	Heterokonta	Pinf	Ensmbl Protist
Thalassiossira pseudonana	Heterokonta	Tpse	Ensmbl Protist 27
Arabidopsis thaliana	Viridiplantae	Atha	Ensmbl Plants 27
Brachypodium distachyon	Viridiplantae	Bdis	Ensmbl Plants 27
Cyanidioschyzon merolae	Viridiplantae	Cmer	Ensmbl Protist 27
Cyanophora paradoxa	Viridiplantae	Cpar	Ensmbl Plants 27
Chlamydomonas reinhardtii	Viridiplantae	Crei	Ensmbl Plants 27
Chlorella variabilis	Viridiplantae	Cvar	Ensmbl Plants 27
Micromonas pusilla	Viridiplantae	Mpus	Ensmbl Plants 27
Ostreococcus tauri	Viridiplantae	Otau	Ensmbl Plants 27
Physcomitrella patens	Viridiplantae	Ppat	Ensmbl Plants 27
Selaginella moellendorffii	Viridiplantae	Smoe	Ensmbl Plants 27
Volvox cartieri	Viridiplantae	Vcar	Ensmbl Plants 27
Bigelowiella natans	Rhizaria	Bnat	Ensmbl Protist 27
Reticulomyxa filosa	Rhizaria	Rfil	Ensmbl Protist 27
Emiliania huxleyi	Haptophyta	Ehux	Ensmbl Protist 27
Guillardia theta	Cryptophyta	Gthe	Ensmbl Protist 27
Bodo saltans	Excavata	Bsal	Ensmbl Protist 27
Leishmania major	Excavata	Lmaj	Ensmbl Protist 27
Naegleria gruberi	Excavata	Ngru	Ensmbl Protist 27
Trypanosoma cruzi	Excavata	Tcru	Ensmbl Protist 27
Trichomonas vaginalis	Excavata	Tvag	Ensmbl Protist 27
Nutomonas longa	Ancyromonadida	Nlon	(Torruella *et al.* 2015)

References

Grau-Bové X, Torruella G, Donachie S et al. (2017) Dynamics of genomic innovation in the unicellular ancestry of animals. eLife, ***6****.*

Hehenberger E, Tikhonenkov D V., Kolisko M et al. (2017) Novel Predators Reshape Holozoan Phylogeny and Reveal the Presence of a Two-Component Signaling System in the Ancestor of Animals. Current Biology, 1–8.

Hellsten U, Harland R, Gilchrist M et al. (2010) charge recombination reaction, it was proposed that structural changes occur- ring in response to electron transfer decrease the free energy gap between P + and Q. Science (New York, N.Y.), ***328****, 633–636.*

Putnam NH, Butts T, Ferrier DEK et al. (2008) The amphioxus genome and the evolution of the chordate karyotype. Nature, ***453****, 1064–1071.*

Richter DJ, Fozouni P, Eisen MB et al. (2018) Gene family innovation , conservation and loss on the animal stem lineage. , ***419411****, 1–43.*

Suga H, Chen Z, de Mendoza A et al. (2013) The Capsaspora genome reveals a complex unicellular prehistory of animals. Nature Communications, ***4****, 2325.*

Torruella G, De Mendoza A, Grau-Bové X et al. (2015) Phylogenomics Reveals Convergent Evolution of Lifestyles in Close Relatives of Animals and Fungi. Current Biology, ***25****, 2404–2410.*

Supplementary Table 8. Summary of protein domains lost at the stem of Metazoa that are the most conserved accross the rest of eukaryotic species (conserved in >50% of the non-Metazoa eukaryotic species used in this analysis).

Protein domains
**Aminoacid and vitamin biosynthesis**
Shikimate_dh_N
DHquinase_I
Anth_synt_I_N
PDT
IlvN
DHquinase_I
Pyridox_oxase_2
GATase_4
**Carbohydrate metabolism**
Pantoate_ligase
Mannosyl_trans3
Glyco_transf_34
**Metal ion binding and metal transport**
CorA
WLM
Zn_clus
SUA5
**Other functions**
Y_phosphatase2
NCA2
Init_tRNA_PT
Lum_binding
BT1
NADH-u_ox-rdase
FUSC_2
VTC
ASL_C
COPI_assoc
ATP_transf
Transferase
**Uknown**
Pho88
HPP
DNAJ-x
PGP_phosphatase
DUF3336
DUF2009
DUF1749
DUF1336
DUF179
DUF8910
DUF1998
DUF1765

Supplementary Table 9. Summary of Opisthokonta species used for the probabilistic analysis of protein domain retention.

**Species**	**Taxonomy**	**Total number of Protein domains**	**BUSCO (%, complete and partial)**
Homo sapiens	Metazoa	5,937	99.7
Mus musculus	Metazoa	5,913	99.7
Xenopus tropicalis	Metazoa	5,213	96.7
Branchiostoma floridae	Metazoa	4,946	99.4
Ciona intestinalis	Metazoa	3,700	82.5
Oikopleura dioica	Metazoa	3,426	97.0
Daphnia pulex	Metazoa	4,205	97.7
Drosophila melanogaster	Metazoa	4,269	99.7
Tribolium castaneum	Metazoa	4,269	97.4
Capitella teleta	Metazoa	4,788	99.0
Saccoglossus kowalevskii	Metazoa	4,000	87.8
Lottia gigantea	Metazoa	4,672	97.7
Trichoplax adhaerens	Metazoa	3,872	98.3
Nematostella vectensis	Metazoa	4,468	97.4
Aiptasia	Metazoa	4,653	97.0
Hydra magnipapillata	Metazoa	3,786	92.7
Mnemiopsis leidyi	Metazoa	3,554	94.1
Acropora digitifera	Metazoa	3,913	80.9
Amphimedon queenslandica	Metazoa	3,413	90.4
Oscarella carmela	Metazoa	3,203	70.9
Sycon ciliatum	Metazoa	4,211	98.7
Codosiga hollandica	Choanoflagellata	3,982	94.7
Helgoeca nana	Choanoflagellata	3,963	95.7
Didymoeca costata	Choanoflagellata	3,950	96.4
Savillea parva	Choanoflagellata	3,955	95.4
Acanthoeca spectabilis	Choanoflagellata	3,958	95.7
Stephanoeca diplocostata	Choanoflagellata	3,724	89.1
Diaphanoeca grandis	Choanoflagellata	3,985	96.4
Salpingoeca dolichothecata	Choanoflagellata	4,132	97.7
Salpingoeca rosetta	Choanoflagellata	3,586	94.1
Salpingoeca roanoka	Choanoflagellata	3,818	96.4
Hartaetosiga balthica	Choanoflagellata	3,510	95.0
Hartaetosiga gracilis	Choanoflagellata	3,470	96.0
Salpingoeca infusionum	Choanoflagellata	3,588	95.0
Monosiga brevicollis	Choanoflagellata	3,230	90.0
Choanoeca perplexa	Choanoflagellata	3,762	96.4
Salpingoeca kvevrii	Choanoflagellata	3,958	96.4
Salpingoeca urceolata	Choanoflagellata	4,058	96.4
Salpingoeca macrocollata	Choanoflagellata	3,994	95.7
Salpingoeca punica	Choanoflagellata	3,788	97.4
Salpingoeca helianthica	Choanoflagellata	3,769	95.7
Mylnosiga fluctuans	Choanoflagellata	3,894	96.7
Pigoraptor vietnamica	Filasterea	3,029	57.4
Pigoraptor chilena	Filasterea	3,706	76.9
Capsaspora owczarzaki	Filasterea	3,565	96.0
Ministeria vibrans	Filasterea	3,385	94.0
Abeoforma whisleri	Teretosporea	3,510	96.03
Creolimax fragrantissima	Teretosporea	3,393	97.7
Pirum gemmata	Teretosporea	3,470	93.4
Sphaeroforma arctica	Teretosporea	3,174	93.1

Sphaerothecum destruens	Teretosporea	3,110	95.4
Chromosphaera perkinsii	Teretosporea	3,692	96.4
Syssomonas multiformis	Teretosporea	4,017	98.3
Corallochytrium limacisporum	Teretosporea	3,197	98.0
Fonticula alba	Discicristoidea	2,399	90.4
Parvularia atlantis.	Discicristoidea	3,571	98.3
Spizellomyces punctatus	Chytridiomycota	3,571	97.4
Batrachochytrium dendrobatidis	Chytridiomycota	3,157	98.3
Mortierella verticillata	incertae sedis	3,545	99.6
Rozella allomycis	Cryptomycota	2,505	87.5
Encephalitozoon cuniculi	Microsporidia	1,017	64.0
Nematocida parisii	Microsporidia	978	51.4
Piromyces sp. E2	Neocallimastigomycota	2,542	73.6
Catenaria anguillulae	Blastocladiomycota	3,088	97.7
Allomyces macrogynus	Blastocladiomycota	3,143	97.0
Rhizophagus irregularis	Glomeromycota	3,441	97.8
Coemansia reversa	Kickxellomycotina	3,018	100
Conidiobolus coronatus	Entomophthoromycota	2,883	92.7
Rhizopus oryzae	Mucoromycotina	3,160	96.7
Gonapodya prolifera	Monoblepharidomycota	3,348	96.8
Coprinopsis cinerea	Basidiomycota	3,308	99.0
Cryptococcus neoformans	Basidiomycota	3,010	98.0
Ustilago maydis	Basidiomycota	3,124	96.7
Neurospora crassa	Ascomycota	3,598	100
Saccharomyces cerevisiae	Ascomycota	2,895	93.7
Schizosaccharomyces pombe	Ascomycota	2,780	86.5

Supplementary Figure 1

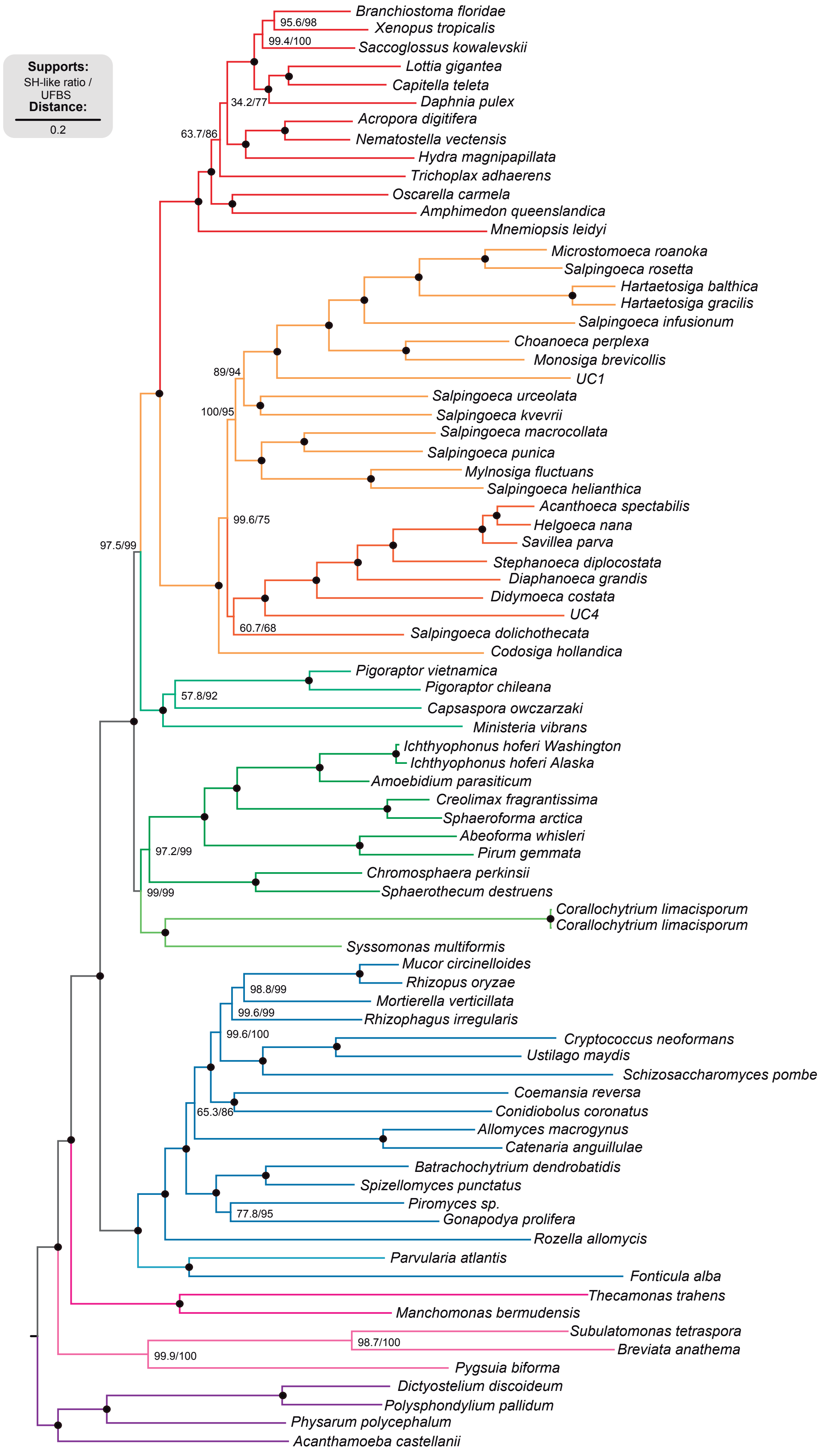

Supplementary Figure 2

A

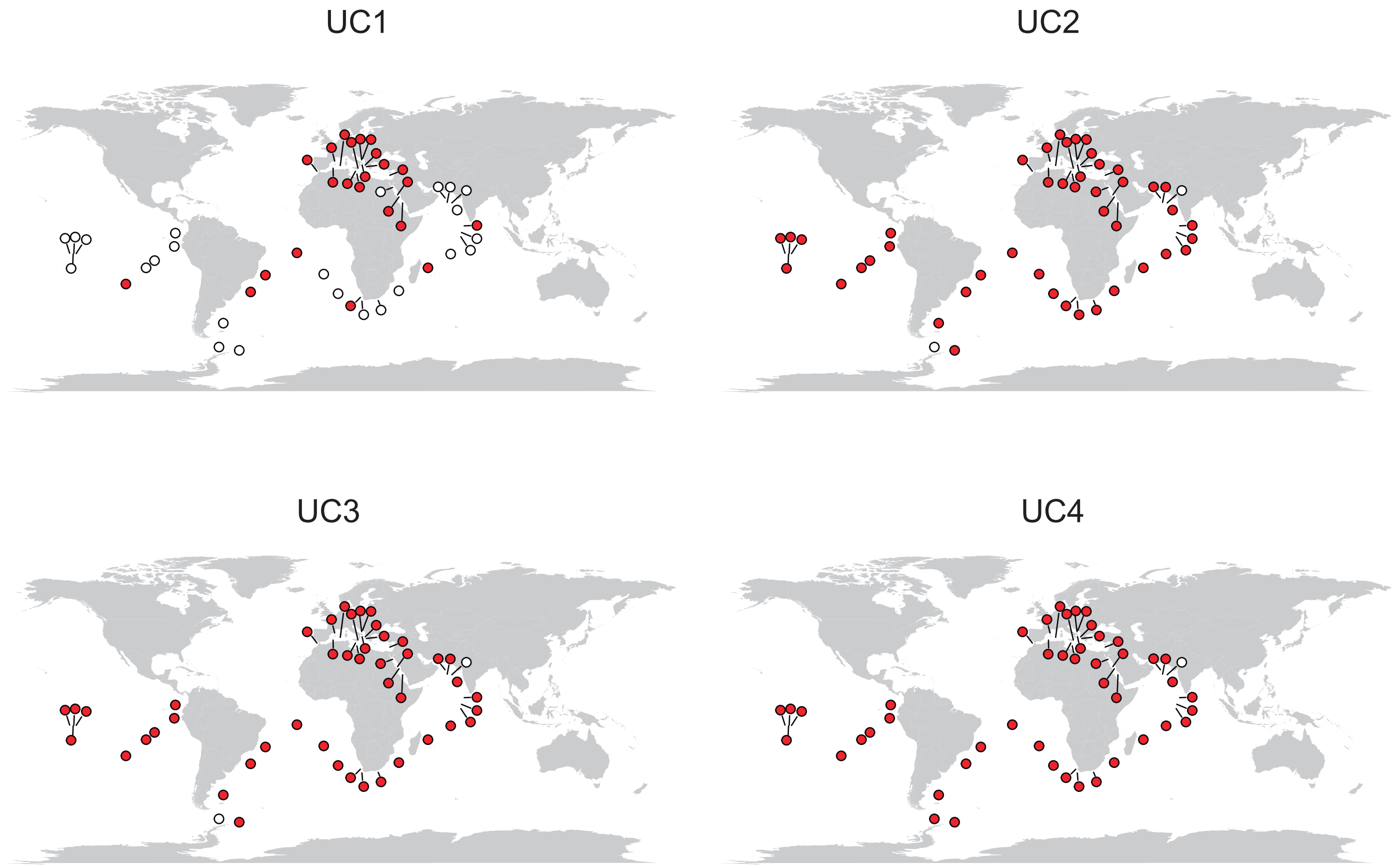

B

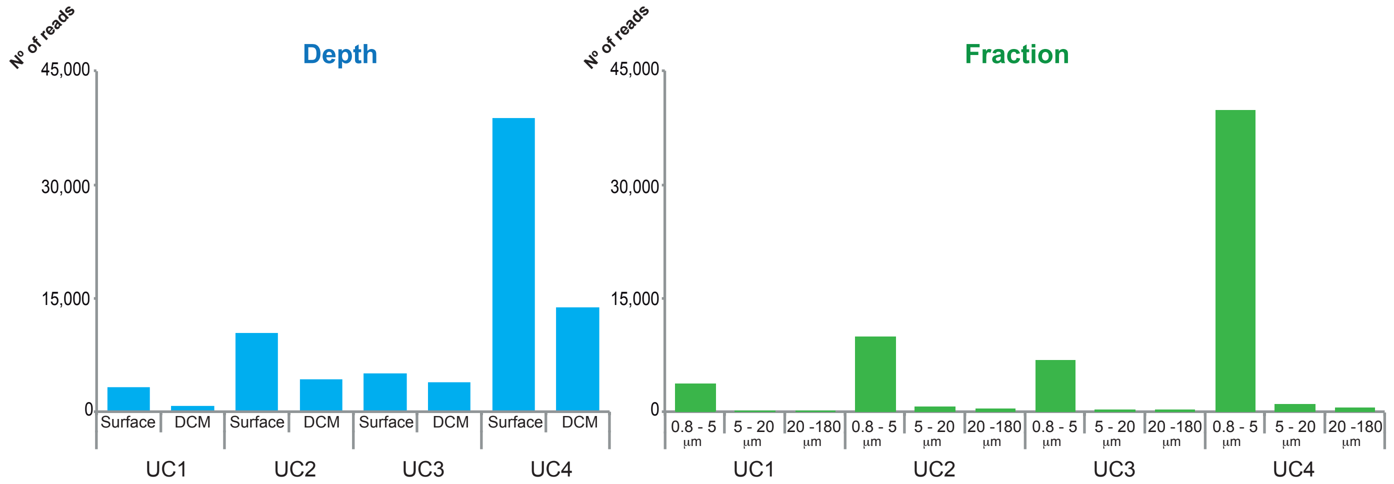

Supplementary Figure 3

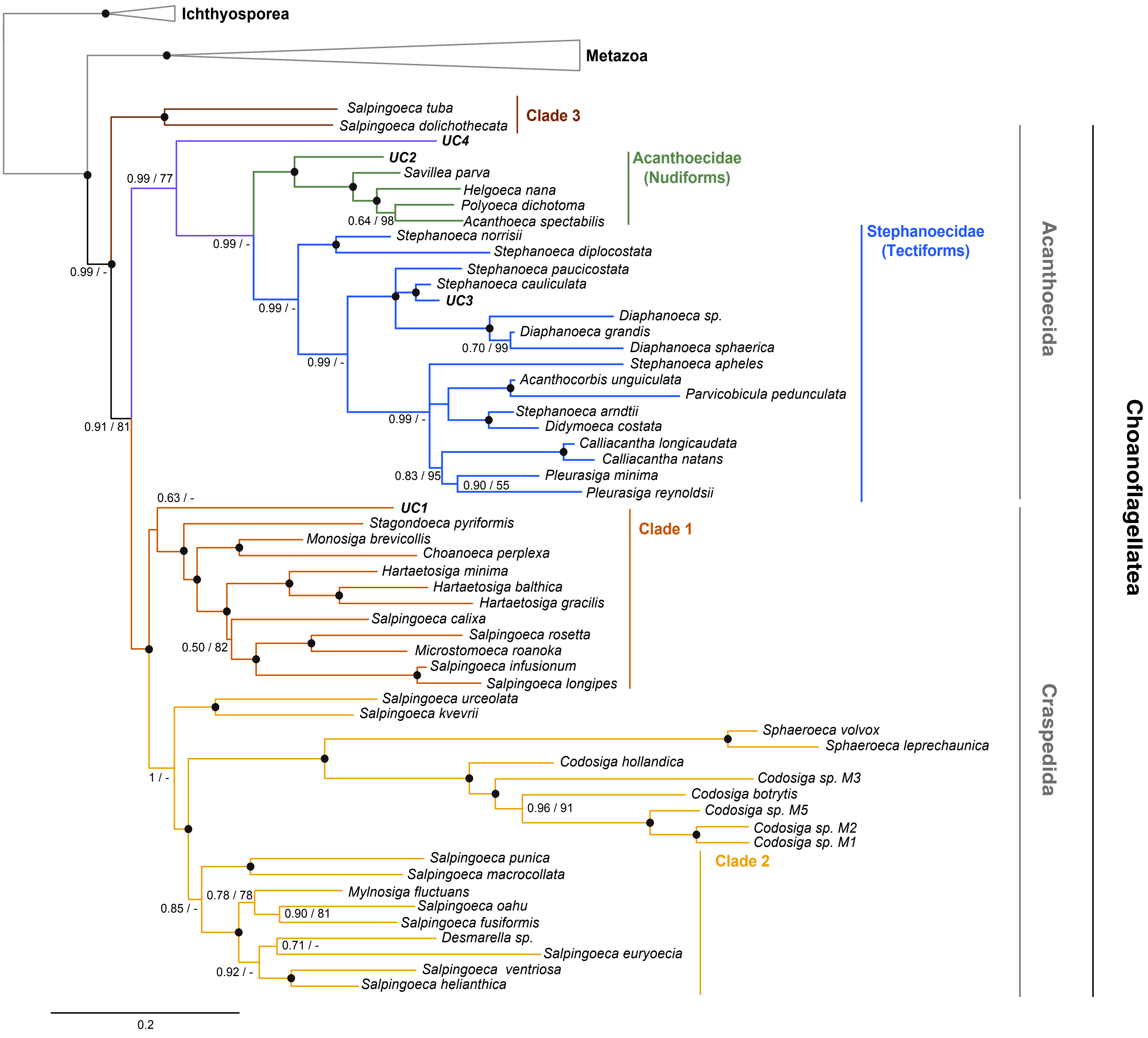

Supplementary Figure 4

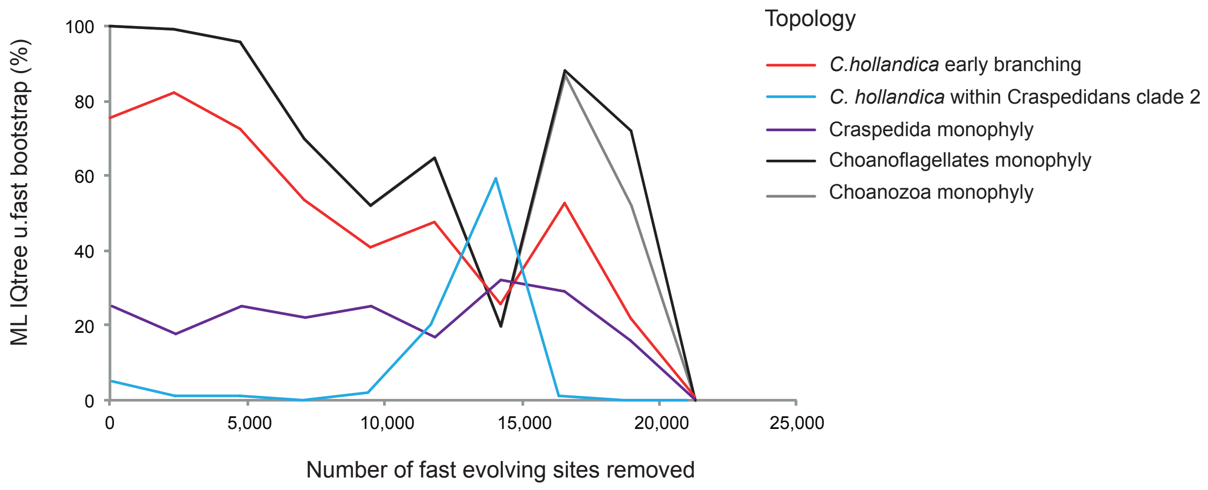

Supplementary Figure 5

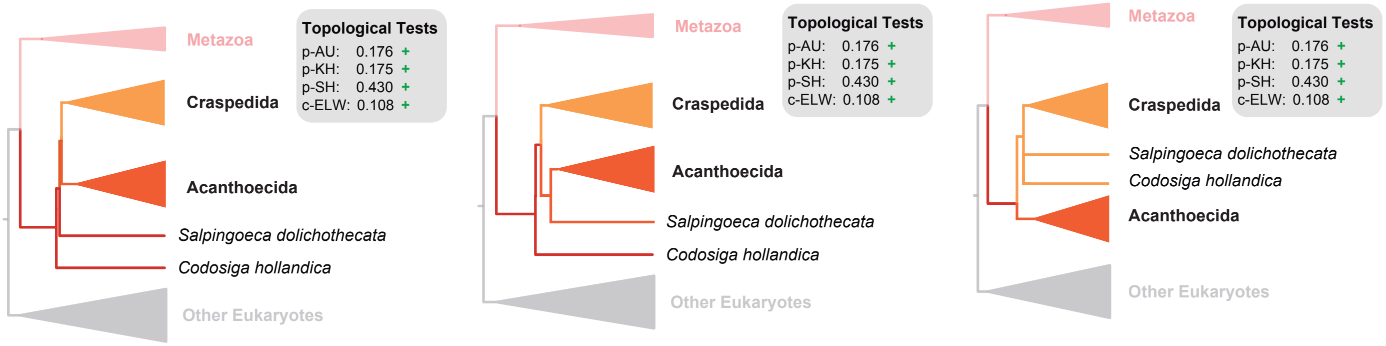

Supplementary Figure 6

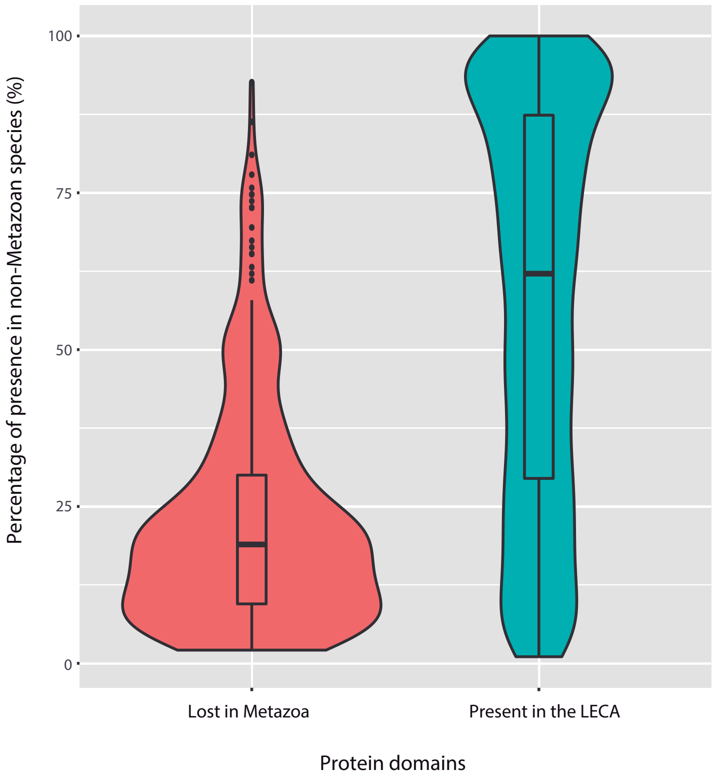

Supplementary Figure 7

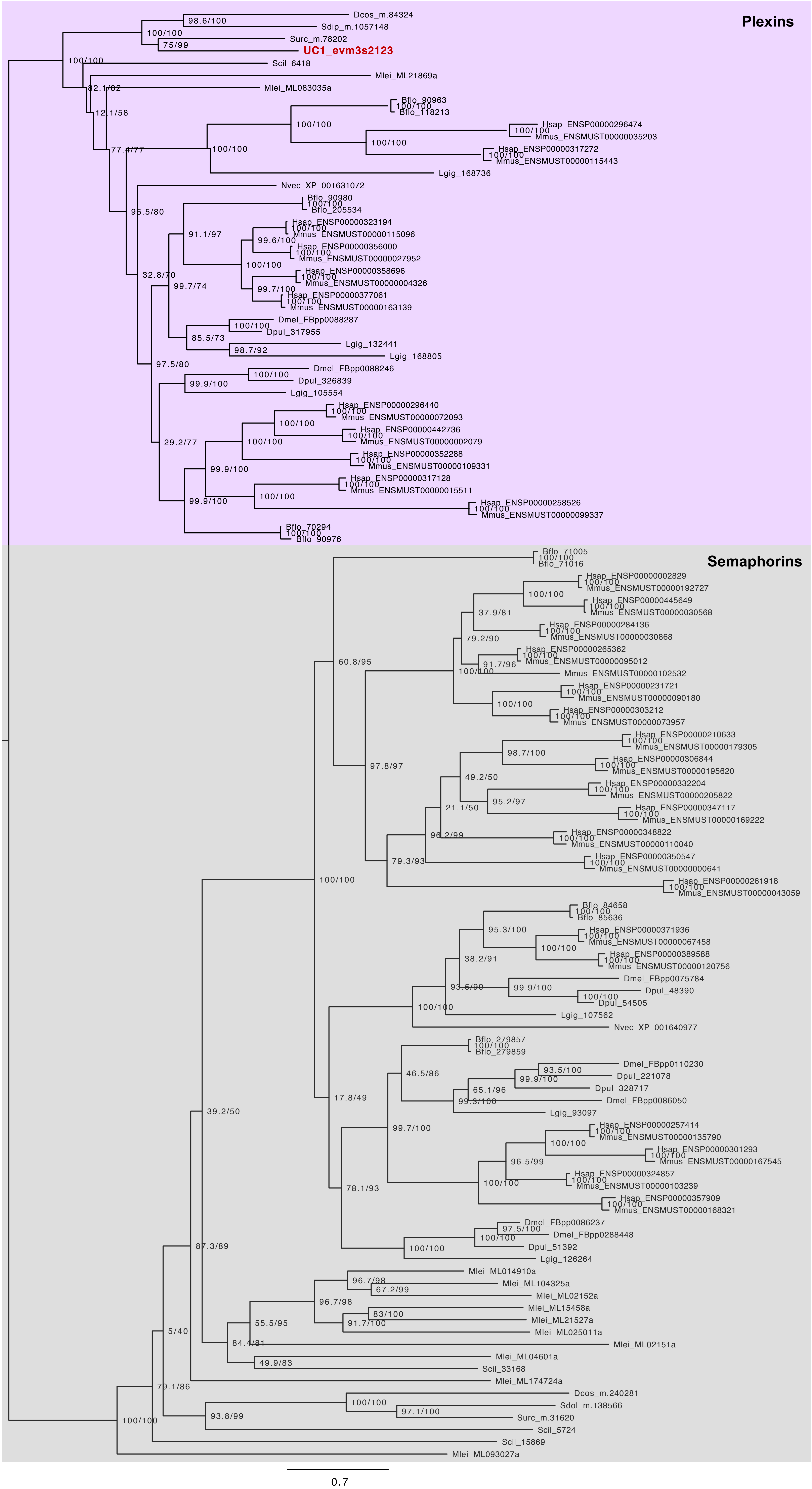

Supplementary Figure 8

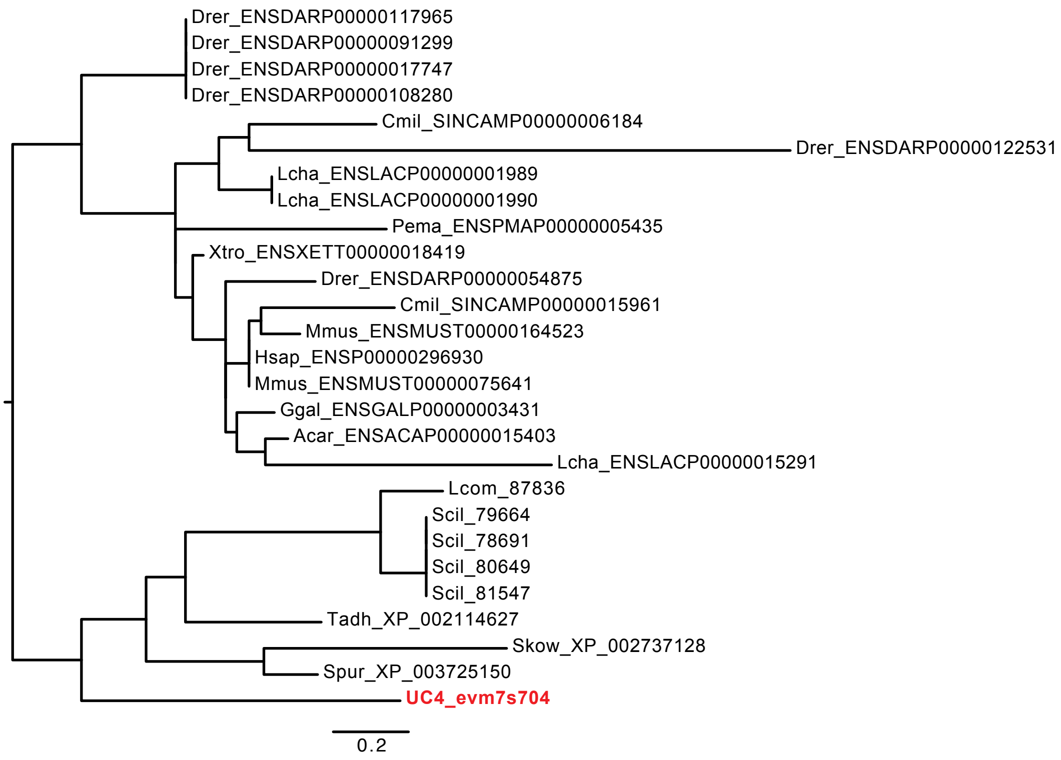

Supplemetary Figure Legends

Supplementary Figure 1. Phylogenomic tree of holozoans, ML inference. Maximum likelihood inference of the UFBS displayed at Figure 3. Calculated with IQtree with LG+R7+C60 model (supports are SH-like approximate likelihood ratio test / UFBS, respectively). Bullets indicate maximum nodal support (100/100).

Supplementary Figure 2. Ecological distribution of our SAGs. (**A**) Geographical location of our SAGs according to the metabarcoding data from TARA oceans expedition (70) (see methods). Red circles mark TARA ocean stations with reads detected from each of our SAGs. White circles represent stations without signal of our SAGs. (**B**) Read distribution according to Depth and size fraction of our SAGs. In blue (left) appears the distribution of reads among different depths: the surface, and the depth clorophyl maximum (DCM). In green (right) it is shown the read distribution according to different size fractions.

Supplementary Figure 3. Eight-gene phylogeny of choanoflagellates. Bayesian inference of an eight-gene phylogeny, adapted from (27) and including the following genes: 18S rDNA, 28S rDNA, hsp90, alpha tubulin, EFL, EF-1A, actin and hsp70 . The choanoflagellate taxa includes our SAGs, the species from (27) and as well the some choanoflagellate taxa described in (103). A summary of taxa is available at Supplementary Table 7. Nodal supports indicates the bayesian posterior probability (right) and the ultra fast bootstrap (UFBS) computed with IQtree (left) in a Maximum Likelihood analysis. The tree produced with ML is available at Figshare (https://figshare.com/s/9ed9c15e93bf4220868e).

Supplementary Figure 4. Fast-evolving site removal of alignment positions sorted by their rates of evolution. Sites were sequentially removed from fastest to slowest positions, 2,310 sites at a time, generating alternative datasets at each step. Ultra-fast bootstrap values were generated and are plotted the ones that support *Codosiga hollandica* as sister-group to the rest of choaonoflagellates (red), the node of the topology that includes *C. hollandica* within Craspedida clade 2 (blue) and the monophyly of Craspedida (purple)**.** As a control are depicted the supports for Choanozoa (grey) and choanoflagellate monophyly (black).

Supplementary Figure 5. Topological test. Representation of the 3 topologies tested: : A) *C. hollandica* as the earliest branching lineage *and S. dolicothecata* sister of the clade formed by the rest of craspedidans and Acanthoecida; B) *S. dolicothecata* branching with Acanthoecida and *C. hollandica* remaining early branching; and C) The classical view in which Craspedida and Acanthoecida are monophyletic. None of them was rejected by any test. Calculated with IQtree with LG+R7+C60 model.

Supplementary Figure 6. **Retention of protein domain lost in Metazoa in other eukaryotic species.** Distribution of the percentage of retention of the protein domains lost in Metazoa in the rest of eukaryotic species (red), compared with the retention of all domains present at LECA (blue).

Supplementary Figure 7. **Phylogeny of the Plexins and Semaphorins.** Maximum likelihood analysis run with IQ-TREE v1.5.1 of the proteins containing the Sema domain, Plexins and Semaphorins. We included the sequences of choanoflagellates, among them the sequence of our SAG UC1 (marked in red), which falls in the Plexin part of the tree. In adition, we selected and important representation of metazoan sequences from our proteome database (Supplementary Table 4 and Figshare

https://figshare.com/s/9ed9c15e93bf4220868e).). Supports are SH-like approximate likelihood ratio test (left) and UFBS, respectively (right) calculated with IQ-TREE v1.5.1.

Supplementary Figure 8. **Phylogeny of the domain NPM1-C.** Maximum likelihood analysis run with IQ-TREE v1.5.1 of the NPM1-C domain, including metazoan representatives of the sequences found in our proteome database (Supplementary Table 4 and Figshare https://figshare.com/s/9ed9c15e93bf4220868e).). Supports are SH-like approximate likelihood ratio test (left) and UFBS, respectively (right) calculated with IQ-TREE v1.5.1
